# The *Gallus gallus* RJF reference genome reveals an MHCY haplotype organized in gene blocks that contain 107 loci including 45 specialized, polymorphic MHC class I loci, 41 C-type lectin-like loci, and other loci amid hundreds of transposable elements

**DOI:** 10.1093/g3journal/jkac218

**Published:** 2022-08-23

**Authors:** Ronald M Goto, Charles D Warden, Takashi Shiina, Kazuyoshi Hosomichi, Jibin Zhang, Tae Hyuk Kang, Xiwei Wu, Marla C Glass, Mary E Delany, Marcia M Miller

**Affiliations:** Department of Molecular and Cellular Biology, Beckman Research Institute, City of Hope, Duarte, CA 91010-3000, USA; Integrative Genomics Core Facility, Beckman Research Institute, City of Hope, Duarte, CA 91010-3000, USA; Division of Basic Medical Science and Molecular Medicine, Department of Molecular Life Science, Tokai University School of Medicine, Isehara, Kanagawa 259-1143, Japan; Division of Basic Medical Science and Molecular Medicine, Department of Molecular Life Science, Tokai University School of Medicine, Isehara, Kanagawa 259-1143, Japan; Department of Molecular and Cellular Biology, Beckman Research Institute, City of Hope, Duarte, CA 91010-3000, USA; Integrative Genomics Core Facility, Beckman Research Institute, City of Hope, Duarte, CA 91010-3000, USA; Integrative Genomics Core Facility, Beckman Research Institute, City of Hope, Duarte, CA 91010-3000, USA; Department of Animal Science, University of California, Davis, Davis, CA 95616, USA; Department of Animal Science, University of California, Davis, Davis, CA 95616, USA; Department of Molecular and Cellular Biology, Beckman Research Institute, City of Hope, Duarte, CA 91010-3000, USA

**Keywords:** MHCY haplotype, chicken RJF reference genome, gene blocks, MHCY class I polymorphism

## Abstract

MHCY is a second major histocompatibility complex-like gene region in chickens originally identified by the presence of major histocompatibility complex class I-like and class II-like gene sequences. Up to now, the MHCY gene region has been poorly represented in genomic sequence data. A high density of repetitive sequence and multiple members of several gene families prevented the accurate assembly of short-read sequence data for MHCY. Identified here by single-molecule real-time sequencing sequencing of BAC clones for the *Gallus gallus* Red Jungle Fowl reference genome are 107 MHCY region genes (45 major histocompatibility complex class I-like, 41 c-type-lectin-like, 8 major histocompatibility complex class IIβ, 8 LENG9-like, 4 zinc finger protein loci, and a single only zinc finger-like locus) located amid hundreds of retroelements within 4 contigs representing the region. Sequences obtained for nearby ribosomal RNA genes have allowed MHCY to be precisely mapped with respect to the nucleolar organizer region. Gene sequences provide insights into the unusual structure of the MHCY class I molecules. The MHCY class I loci are polymorphic and group into 22 types based on predicted amino acid sequences. Some MHCY class I loci are full-length major histocompatibility complex class I genes. Others with altered gene structure are considered gene candidates. The amino acid side chains at many of the polymorphic positions in MHCY class I are directed away rather than into the antigen-binding groove as is typical of peptide-binding major histocompatibility complex class I molecules. Identical and nearly identical blocks of genomic sequence contribute to the observed multiplicity of identical MHCY genes and the large size (>639 kb) of the Red Jungle Fowl MHCY haplotype. Multiple points of hybridization observed in fluorescence in situ hybridization suggest that the Red Jungle Fowl MHCY haplotype is made up of linked, but physically separated genomic segments. The unusual gene content, the evidence of highly similar duplicated segments, and additional evidence of variation in haplotype size distinguish polymorphic MHCY from classical polymorphic major histocompatibility complex regions.

## Introduction

In the genome of chickens, there is a gene cluster named MHCY. First identified in Southern blots, MHCY was originally named *restriction fragment pattern-Y* (*Rfp-Y*; Briles *et al.* 1993). It was later renamed *Y* (Miller *et al.* 2004) and is often described as MHC-*Y* (Miller and Taylor 2016), which is written here as MHCY. MHCY is known to contain major histocompatibility complex (MHC) class I-like (sometimes named YF), MHC class Iiβ, and c-type lectin-like genes (Briles *et al.* 1993; Miller *et al.* 1994; Afanassieff *et al.* 2001; Rogers *et al.* 2003). The MHCY and MHCB, the latter being the canonical MHC in chicken, are located on the q-arm of chicken chromosome 16 (GGA16) with MHCB distal to MHCY (Delany *et al.* 2009). PO41 repetitive sequences in tandem and inverted orientations separate the 2 regions (Solinhac *et al.* 2010). Highly frequent meiotic recombination results in MHCY and MHCB haplotypes assorting independently, as if the 2 regions were located on different chromosomes (Briles *et al.* 1993; Zhang, Goto, Honaker *et al.* 2022). Proximal to MHCY is the single nucleolus organizer region (NOR) in chicken (Delany *et al.* 2009), which consists of about 150 tandem repeats per chromosome of the ribosomal RNA gene cluster (encoding 18S, 5.8S, 28S rRNA) in chicken. Proximal to the NOR, and in linkage with MHCY, is a region of olfactory receptor and scavenger receptor genes (Miller *et al.* 2014).

Obtaining sequence for the MHCY gene region was considerably delayed relative to sequencing of many other portions of the chicken genome including MHCB. The unusually high density of repetitive sequences and gene family members made it impossible to assemble short-read sequencing data for the MHCY region. Sequences of cosmid and other small insert clones provided a glimpse of the unusual nature of MHCY and of the special features of MHCY class I gene sequences (Guillemot *et al.* 1988; Afanassieff *et al.* 2000, 2001; Rogers *et al.* 2003; Miller and Taylor 2016). These studies showed that MHCY was not simply another classical MHC. MHCY-encoded class I molecules lack residues known to be critical for anchoring peptide antigens in the class I antigen-binding groove (Afanassieff *et al.* 2001). No evidence was found for peptide antigen in the binding groove of MHCY class I molecules in a set of unpublished experiments that were companion to the identification of peptides bound by MHCB class I molecules that served as controls (Sherman *et al.* 2008). The MHCY class I antigen-binding groove is too narrow to accommodate peptides (Hee *et al.* 2010). Indeed, lysophospholipids have recently been identified by mass spectrometry as candidate ligands (Gugiu, Goto, Bhattacharya, Delgado, Dalton, Balendiran, and Miller, submitted). With the development of sequencing technology that dependably provides long-read sequences (Eid *et al.* 2009), it has been possible to obtain sufficient data to define MHCY gene region sequence cloned within BACs. As described below, the region is far more complex than initially considered. Genetic variability is present at several levels.

Evidence that there may be an association of MHCY genotype with immune responses and disease incidence is emerging. Initial disease challenge trials, all of which were done with small sample sets, provided mixed evidence for a contribution of MHCY in the growth of tumors associated with oncogenic Marek’s disease and Rous sarcoma viruses (Bacon *et al.* 1996; Wakenell *et al.* 1996; Vallejo *et al.* 1997; LePage *et al.* 2000; Pinard-van der Laan *et al.* 2004; Praharaj *et al.* 2004). More recent experiments with larger sample sets from the Virginia Tech and Wageningen University lines selected over many generations for high and low antibody responses, provided evidence for significant heritable association between MHCY haplotype and antibody titer (Zhang, Goto, Honaker *et al.* 2022). An association between MHCY haplotype and colonization of chickens by *Campylobacter* has been noted recently in a backcross [(Line 6(1) × Line N) × Line N] population (Zhang, Goto, Psifidi *et al.* 2022). MHCY class I genes are among genes upregulated in response to immunological challenges (Connell *et al.* 2012; Geng *et al.* 2015; Wu *et al.* 2015). These findings suggest that further investigation of the MHCY region and its genetic variability is important for understanding differences among individual animals in their capacity to respond to disease challenges.

This study was undertaken to advance understanding of the organization of the MHCY region. The overall aim of our studies has been to define MHCY region genetic variability and to gain insight into how MHCY haplotype differences might support variability in immune responses among chickens. BAC cloning and single-molecule real-time sequencing (SMRT) has allowed large sections of sequence for the large MHCY haplotype in the chicken Red Jungle Fowl (RJF) reference genome to be assembled, annotated, and analyzed.

## Methods

### BAC clone isolation and sequencing

Array filter sets for the *Gallus gallus* RJF reference genome BAC clone libraries were provided by the USDA NRSP8 National Animal Genome Research Program and obtained from Jerry Dodgson (Michigan State University). All libraries were made with DNA from a single, highly inbred Line UCD001 female, #256. MHCY testing of multiple Line UCD001 individuals at City of Hope suggested that the UCD001 line is fixed for a single MHCY haplotype, which we designate here as the RJF MHCY haplotype. The array filters were spotted with the clones in 3 libraries (partial digests with *Bam*HI, *Eco*RI, and *Hind*III) made at Texas A&M University (TAMU), College Station, TX (Lee *et al.* 2003) and in a fourth library (CHORI-261, *Eco*RI partial digest) made at the BACPAC Resource Center, Children’s Hospital Oakland Research Institute, Oakland, CA). The filter sets were screened with *163/164f* (GenBank AF493428), a probe specific for MHCY class I loci, and with *439/432*, a probe specific for MHCY class IIβ loci (GenBank DQ007238.1). Probes were randomly primed with ^32^P, and the filter sets were hybridized under the same conditions as for Southern hybridizations made with these probes (Briles *et al.* 1993; Afanassieff *et al.* 2001). Candidate clones identified in the filter set hybridizations were obtained from TAMU and the BACPAC Resource Center and verified in Southern hybridizations to be MHCY positive. BAC clones were propagated in DH10B *Escherichia coli* and grown in LB with chloramphenicol (30 µg/ml) selection. BAC DNA was isolated using Qiagen Large-Construct Kit (cat. no.12462) with yields of 7–15 µg of highly purified BAC DNA from 0.5-l preps. In preparation for PacBio SMRT (Pacific Biosciences, Menlo Park, CA) sequencing, purified BAC DNA was prepared following standard methods (see Supplementary Methods).

Seven BAC clones representing MHCY are included in this study (Table 1). CHORI-261-173o1 and TAM033-JF256-H3-34j16 (henceforth known as 173o1 and 34j16), were first Sanger sequenced at Tokai University School of Medicine and later resequenced with SMRT sequencing at City of Hope. Five additional MHCY clones, CHORI-261-190m7, CHORI-261-102b15, CHORI-261-1o23, TAM032-JF256-RI-19d16, and TAM032-JF256-RI-58f18 (henceforth known as 190m7, 102b15, 1o23, 19d16, and 58f18) were also SMRT sequenced. An eighth MHCY clone identified, TAM031-JF256-BI-66a9, is not included because it was only Sanger sequenced and it corresponds to an area entirely within 173o1. To further test the quality of sequence assemblies, additional sequence data were collected with Illumina sequencing methods and with PacBio CCS HiFi reads (see Supplementary Methods).

**Table 1.** BAC and fosmid clones within the MHCY contigs.

Contig	Contig size (bp)	Clones within Contig	BAC vector	size (bp)	SMRT	Sanger	Illumina	Gene content	GenBank number
1	412,370	CHORI-261-R1-190m7	pTARBAC2.1	253,430	Yes	No	Yes	NOR and MHCY	MW583602
		CHORI-261-R1-102b15	pTARBAC2.1	102,137	Yes	No	No	NOR and MHCY	MW583600
		CHORI-261-R1-1o23	pTARBAC2.1	77,997	Yes	No	No	NOR and MHCY	MW583598
		CHORI-261-R1-173o1^a^	pTARBAC2.1	221,406	Yes	Yes	Yes	MHCY	MW583601
		TAM032-JF256-RI-19d16	pECBAC1	158,940	Yes	No	No	MHCY	MW583599
2	148,501	TAM032-JF256-RI-58f18	pECBAC1	148,501	Yes	No	No	MHCY	MW583604
3	138,921	TAM033-JF256-H3-34j16	pBeloBAC11	138,921	Yes	Yes	Yes	MHCY	MW583605
4	45,013	J_AE-174A8	Fosmid	36,072	No	No	Yes	MHCY	AC270418.1
		J_AD-484G5	Fosmid	36,027	No	No	Yes	MHCY	AC270441.1

Total Overall =744,805 bp (NOR 106,160 bp). The overall total for MHCY is 638,645 bp with 306,210 bp from Contig1 and 332,435 bp from Contigs 2-4.

a Found to match GenBank AC275299.1 (J_AA173O01)

### Sequence assembly for clones and contigs, validation, and annotation of genes and repeat sequences

The assembled contig sequences were initially analyzed with GENSCAN to identify potential coding sequences (Burge and Karlin 1997). Additional gene candidate sequences were added as they became apparent. Proposed intron/exon boundaries were evaluated using RJF RNA Seq libraries available in Chickspress (McCarthy *et al.* 2019). Gene models were ranked using normalized counts per junction and graded for the fraction of junctions covered. Sequence differences among members of gene families were defined based on visual inspection and sequence variability revealed in CLUSTALW (CLC Genomics Workbench 6.0.5, www.clcbio.com) alignments. The ribosomal RNA genes were annotated using KT445934 as a guide (Dyomin *et al.* 2016). The sequences were analyzed for retroelements, DNA transposons, small RNA, satellites, simple repeats, and low complexity sequences using RepeatMasker (Smit, AFA, Hubley, R & Green, P. *RepeatMasker Open-4.0*. 2013–2015; http://www.repeatmasker.org) with chicken RepBase annotations (Bao *et al.* 2015, downloaded 2017 June 4). Tandem Repeat Finder (http://tandem.bu.edu/trf/trf.html, Benson 1999) was used to define tandem repeats. For details of sequence assembly, and validation see Supplementary Methods.

### Acquisition of MHCY class I cDNA sequences

All studies with live animals were covered by active Animal Care Protocols. cDNA clones were made using RNA isolated from liver tissue from a UCD001 individual collected into Qiagen Allprotect Tissue Reagent. Cornell Lines N fertile eggs were obtained from the USDA ARS Avian Disease and Oncology Laboratory (East Lansing, Michigan) and hatched at City of Hope. Liver and spleen tissue were collected into RNAlater (Ambion) from 1-week-old chicks. Small tissue slices (∼10–50 mg) were homogenized, and RNA was extracted with RNeasy (Qiagen) following the manufacturer’s protocol. Two microgram aliquots of total RNA were used in 20 µl reverse transcriptase reactions (SuperScript II, Invitrogen) with either random or oligo-dT priming. Typically, 0.5 µl of this reaction was used as the template for PCR amplification with MHCY class I-specific primers: 5ʹ-primer (#811) ATGGGTCCGAGCGAGGTGGTG and 3ʹ-primer (#812) TCAGATGGAAGGTTCACTTC and KOD high fidelity DNA polymerase (EMD Millipore, Burlington, MA), a highly efficient proof-reading enzyme. Products of the PCR reaction were separated on a 1.5% TAE agarose gel and bands of the expected size (∼1,000 bp) were cut from the gel. PCR products were recovered by Glass Milk (Bioworld) purification and ligated into the *Eco*RV site of pBluescript II using T4 ligase (NEB) in overnight reactions at 4°C. DNA purified with Qiagen minipreps was submitted for sequencing. Sequences were assembled and analyzed with Vector NTI.

### Analysis of the distribution of polymorphic residues in MHCY class I molecules

Amino acid sequences predicted in the α1 and α2 domains of MHCY class I loci and of classical, peptide-binding HLA class I were aligned and analyzed to define positions of highly variable residues (Wu and Kabat 1970). Positions with variability index (VI) scores of 6 or greater were considered polymorphic. Fifteen MHCY cDNA sequences were analyzed. These include: (1) YF1*7.1 (AF218783), (2) 10 RJF MHCY1 sequences from Types a-j, and (3) 4 MHCY1 cDNA clones from other haplotypes in Cornell Line N. The HLA sequences included 25 HLA-A and HLA-B sequences available in GenBank. All GenBank entries were verified in the IMGT database (https://www.imgt.org/) as representing the HLA loci named. PyMOL (2010 Schrodinger, LLC) was used to produce structures for illustrating the distribution of polymorphic positions on structures of the MHCY class I (PDB ID 3P77) and HLA (PDB ID 1HLA) class I molecules.

### Patterns of nucleotide substitutions

A neighbor-joining phylogenetic tree was generated using the α1 and α2 domain sequences of the 15 MHCY loci with typical MHC class I structure. Two branches in which genes from the RJF haplotype and other haplotypes were present were selected to complete the comparison with the nucleotide substitutions occurring in classical, peptide-binding loci. Sequences from 2 HLA loci were used to represent classical loci. The sequences were analyzed for patterns of nucleotide substitution using PAML (Bitarello *et al.* 2016). Full details of the analysis are available in Supplementary Methods.

### Southern blot analysis

*Taq*1-digested genomic DNA (10 µg) samples were electrophoresed into 1% agarose gels buffered with 0.8% Tris/Borate/EDTA buffer, pressure blotted into Gene Screen hybridization membranes, and immobilized by UV cross-linking. After blocking, the filters were hybridized overnight with ^32^P-labeled *163/164f* (GenBank AF493428), washed, and autoradiograms produced with a Quanta III intensification screen (Briles *et al.* 1993; LePage *et al.* 2000; Afanassieff *et al.* 2001). MHCY haplotypes originate from previously typed individuals: RJF (Line UCD001, #256, UC Davis; International Chicken Genome Sequencing Consortium 2004), Y1-Y3 (members of A436 and A438 families, Northern Illinois U), Y4-Y6 (disomic members of the Cornell trisomic line; Bloom and Bacon 1985; Miller *et al.* 1996), and Y7, Y8, and Y5/9 (Cornell Lines N and P; Pharr *et al.* 1997).

### Fluorescence in situ hybridization

A whole spleen was collected from a single RJF (Line UCD-001) rooster. Chromosome spreads from untreated spleen cells (no mitogen or Colcemid) were made on glass slides and fixed following standard procedures (Rodionov *et al.* 2002; Romanov *et al.* 2005; Delany *et al.* 2009). To identify the NOR, a 3-kb fragment from the 5ʹ-region of the 18S-5.8S-28S ribosomal gene repeat, the external transcribed sequence (ETS), was cloned into pSport and directly labeled with Spectrum Red (Abbott Molecular Inc.) by nick translation (Delany and Krupkin 1999). The MHCY probe [34j16 (Contig 3)] was Digoxigenin (DIG) conjugated and visualized with an anti-DIG FITC (green) antibody (Delany and Krupkin 1999). Slides were counterstained with 4′,6-diamidino-2-phenylindole (DAPI). For nuclei to be selected for inclusion in the analysis, each nucleus was required to exhibit 2 NOR fluorescence in situ hybridization (FISH) signals (signifying each GGA16 chromosome), each NOR signal had to have at least one adjacent MHCY FISH signal, there could be no overlap of the signals (proximity acceptable, but with the signals sufficiently distinct) originating from the 2 copies of GGA16. There could be no major overlap between neighboring nuclei. For MHCY FISH signal counting, possible merged signals were counted as one unless distinct units were clear.

### Dot plots

Dot plots were generated in 2 sequence BLASTn Megablast searches. Contig 1 served as the base sequence for all comparisons. Labels indicating the positions of individual genes were then added manually.

### Sequence deposition

Raw data and finished sequences were submitted to GenBank (https://www.ncbi.nlm.nih.gov/genbank/submit/). SRA accession numbers are: SRR17888437–SRR17888450. Fully annotated sequences for the 3 BAC contigs are: Contig1 (MW583603), Contig2 (MW583604), and Contig 3 (MW583605). Sequences for the individual BAC clones making up Contig1 are: BAC1o23 (MW583598), BAC19d16 (MW583599), BAC102b15 (MW583600), BAC173o1 (MW583601), and BAC190m7 (MW583602). Annotated sequence for Contig 4 is deposited in the GenBank Third Party Annotation (TPA) database (BK059164). Portions of the genomic sequences reported here were shared in 2017 March to aid in assembly of the chicken reference genome GRCg6. Contigs 1-3 contributed to NCBI RefSeq updates in advance of the release of the GRCg7b and GRCg7w assemblies on 2021 April 9. Deposited sequences for MHCY class I cDNA sequences from RJF are Type a (*YF8*, *YF17*, and *YF21*) (MW423627), Type b (*YF34*) (MW423628), and Type c (*YF37*) (MW423629), and an additional RJF cDNA from an unidentified RJF YF locus (MW423630). The cDNA clones from Cornell Line N are: 5.1 (MW917156), 5.2 (MW917157), 8.1 (MW917158), and 8.2 (MW917159).

## Results

### BAC clone and fosmid sequences sort into 4 contigs representing MHCY and NOR

**Fig. 1.**
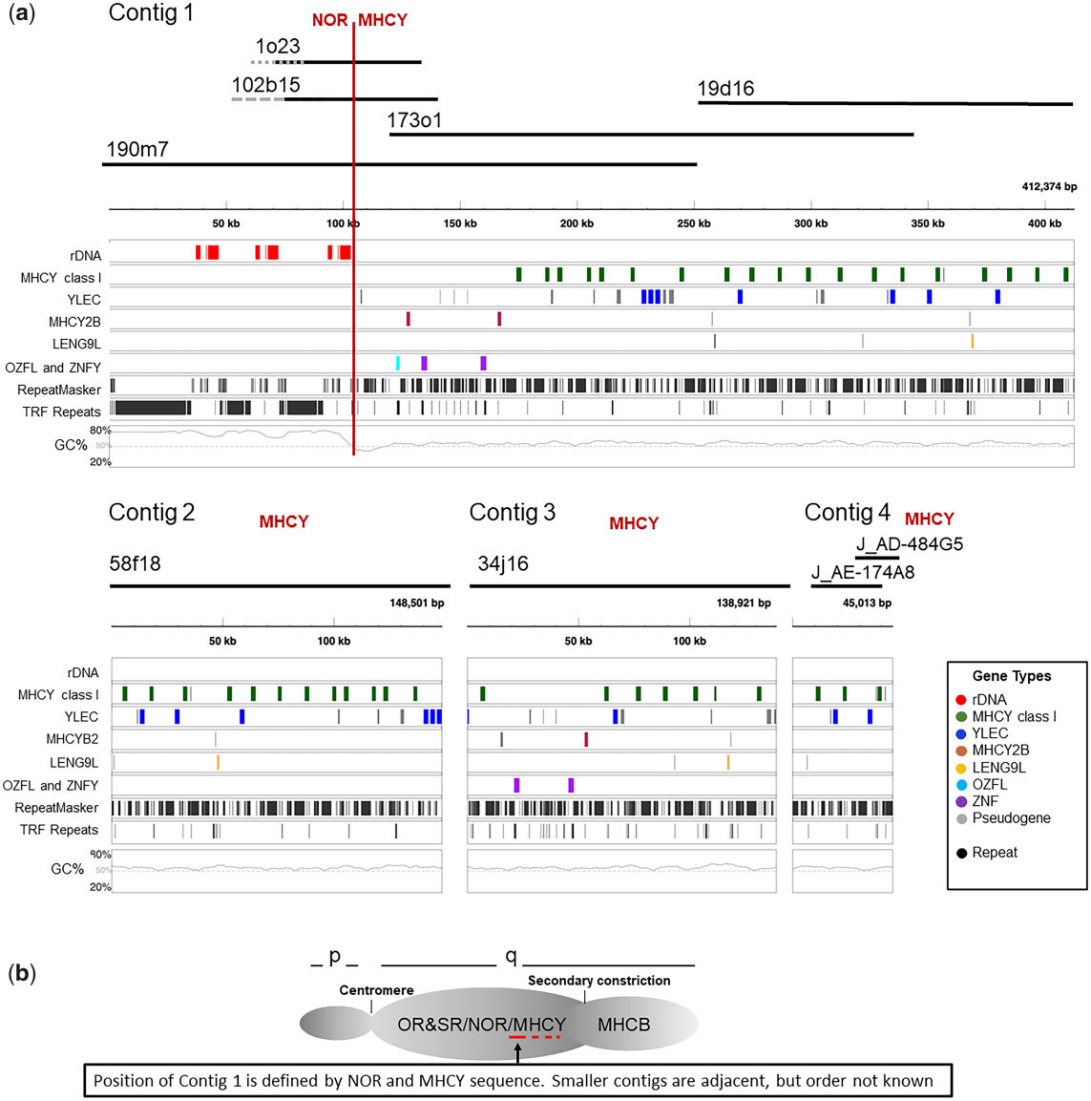
Overview of the organization of the MHCY gene region on GGA16 in the chicken RJF reference genome. a) Sequence data from 7 BAC and 2 fosmid clones form 4 contigs containing 107 genes and hundreds of repetitive elements within the RJF MHCY haplotype. Also present are 3 ribosomal RNA gene sequences that allow the position of MHCY relative to the NOR to be precisely defined. b) Diagram showing position of the contigs relative to other loci previously mapped to GGA16.

Screening of the available RJF BAC libraries with probes specific for MHCY class I and class II genes provided 7 BAC clones (Table 1). All were PacBio SMRT sequenced. Full-length assemblies were achieved for each clone and were carefully verified for accuracy during assembly of the contigs. Coverage is extensive for each (Supplementary Fig. 1). Contig 1 contains the sequences of 5 BAC clones 190m7, 19d16, 173o1, 1o23, and 102b15 (Fig. 1a). Three rRNA gene arrays and adjacent intergenic spacer sequences define the left margin of Contig 1. The 3 rRNA gene arrays are highly similar to each other and to the first sequence published for a complete chicken rRNA gene cluster (Dyomin *et al.* 2019). The inclusion of 2 clones (1o23 and 102b15) into Contig 1 required particular care. Both contain MHCY and NOR region sequence. The 1o23, 102b15 clones have identical sequence at their 3ʹ-ends (containing MHCY and a portion of the immediately adjacent NOR). Clones 1o23 and 102b15 match 190m7 and 173o1 perfectly over this region. At their 5ʹ-ends 1o23 and 102b15 differ from each other and from 190m7 (regions of mismatch are noted by 2 types of dashed lined in Fig. 1a). In the mismatch region, both clones have sequence typical of the NOR but do not align perfectly with the NOR sequence in 190m7. We do not know for certain the basis of the mismatch, but the chicken genomic sequence originally cloned may have been altered during early propagation. Although generally stable, DNA inserted in BAC vectors may undergo deletions during propagation. Large deletions have been observed to occur in tandemly repeated DNA elements early in propagation (Song *et al.* 2001; Kanda *et al.* 2011). In this instance, 1o23 and 102b15 may have undergone deletions in which segments of the NOR were “looped out” resulting in smaller BAC clones in which noncontiguous portions of the NOR with similar, but not identical sequence became juxaposed. Both clones are significantly smaller (79 and 102 kb, respectively) than 173o1 (221 kb) and 190m7 (253 kb) isolated from the same pTARBAC2.1 library.

The NOR and MHCY are closely positioned. The 3ʹ ETS of the third rDNA gene array (shown in Fig. 1) is located at 103,101–103,436 in Contig 1. Just 2,725 bp following, at 106,161–106,385 bp, is an ERV1 GGLTR-7-int LTR sequence, a repetitive element commonly found within the MHCY region sequence and never found within the NOR sequence. There is little evidence for specialized sequence insulating the MHCY and NOR boundary, except for a 39 bp distinctive, repetitive mark at 105,743–105,757 bp with the core sequence of CCCTCCTCCCCTCCT. This sequence is found nowhere else within the sequence reported here.

Additional portions of MHCY contained in clones 58f18 and 34j16 provide Contigs 2 and 3. Contigs 2 and 3 are distinct from each other and from Contig 1 and Contig 4. Contig 4 is a small contig that was defined by 2 fosmid clone sequences, J AD-484G5 and J AE-174A8, previously deposited in GenBank (AC270441.1 and AC270418.1) as part of an earlier study (Bellott *et al.* 2017).

The 4 contigs cover a total of 744,805 bp of chicken GGA16 in which MHCY (638,645 bp) and ribosomal RNA genes of the NOR (106,160 bp) are present. For simplicity, in Fig. 1a the contigs are ordered by size. Only Contig 1 can be positioned with certainty because it contains MHCY and NOR sequence (Fig. 1b). The relative order of the Contigs 2, 3, and 4 is not known.

### Representation of the RJF haplotype within the sequence data

**Fig. 2.**
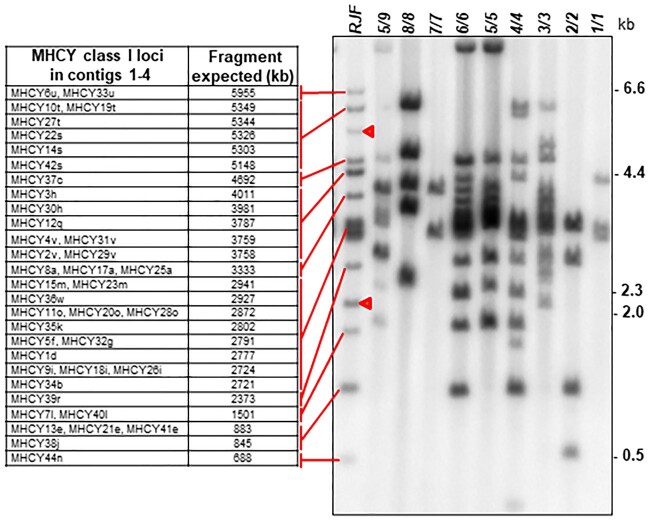
Southern hybridization used to evaluate coverage of the RJF haplotype by sequences within the 4 contigs and to illustrate the relative complexity of the RJF MHCY haplotype. All *Taq*1 fragments predicted in the contig sequences (*left*) can be assigned to restriction fragments revealed in a southern blot of *Taq*1-digested RJF DNA (*right*) hybridized with the MHCY class I-specific probe, *163/164f*. Bars and lines are guides to matches between predicted and the restriction fragments observed in the RJF southern blot. Two RJF restriction fragments have no matches (arrowheads) indicating that portions of the RJF haplotype are not with Contigs 1–4. The restriction fragment patterns for other MHCY haplotypes included in the southern blot show that MHCY haplotypes vary widely in complexity.

The RJF haplotype is one of the most complex MHCY haplotypes observed in southern blots (Fig. 2). At least 12 *Taq*1 restriction fragments make up the RJF MHCY pattern. To evaluate the extent to which the sequence in the 4 contigs represents the RJF haplotype, we defined the sizes of predicted *Taq*1 restriction fragments present within the contig sequences that would be expected to hybridize with the *163/164f* probe and compared these sizes with the sizes of bands actually hybridizing with *163/164f* in the southern blot of *Taq*1-digested RJF DNA. All the predicted fragments could be matched with fragments in the blot. But 2 fragments within the blot are without counterparts in the restriction fragments defined by the contig sequences. This indicates that a portion of MHCY was not captured within the isolated clones. A RJF MHCY class I cDNA clone (GenBank MW423630) for which no gene is identified, provides further evidence that that not all the RJF MHCY haplotype has been captured in the BAC clones.

### Mapping MHCY to GGA16 in the RJF karyotype

**Fig. 3.**
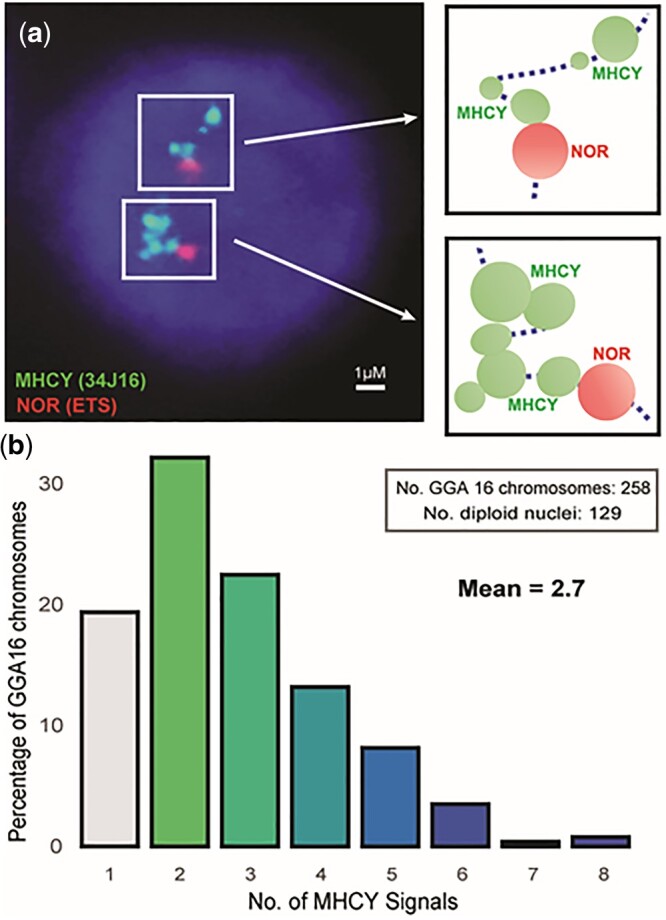
FISH reveals multiple discrete points of MHCY probe hybridization on RJF chromosome 16 (GGA16). a) The MHCY class I probe hybridizes in FISH at multiple discrete MHCY points in the extended chromatin of RJF GGA16. The 2 copies of GGA16 are identified by hybridization of the ribosomal rDNA ETS (external transcribed spacer) pSport probe to the NOR (*left*). Multiple points of hybridization with the MHCY probe (34J16, aka Contig3) provide evidence for the separation of the MHCY region into multiple discreet units, one of which is immediately adjacent to the NOR. A diagrammatic guide to the hybridization is provided (*right*). b) To further define MHCY FISH hybridization on RJF GGA16, points of MHCY probe hybridization were counted on multiple chromosomes. Most chromosomes showed multiple loci of MHCY probe hybridization (80.6%) with the number of loci on individual chromosomes ranging from 2 to 8. Single MHCY signals were less frequent (19.4%). These may represent single points of hybridization or combined multiple hybridization points providing merged FISH signals.

To gain insight into why the genomic sequence obtained for the RJF MHCY haplotype failed to assemble into a single contig, FISH preparations were made using interphase nuclei so we could capture FISH images of RJF chromosomes in extended confirmation. An NOR ETS probe Fig. 3a) was used to identify GGA16. The 34j16 BAC clone (Contig 3, DIG conjugated and visualized with an anti-DIG FITC) was used to identify MHCY (Fig. 3a). The FISH images revealed multiple discrete points of MHCY hybridization along GGA16. One point of MHCY hybridization was always observed to be immediately adjacent to the NOR. This is consistent with the observation that MHCY and NOR sequences are separated by less than 3 kb (Fig. 1a). Multiple, well-separated points of MHCY hybridization were also observed. The mean number of MHCY hybridization points observed among 258 copies of Chr16 scored was 2.7 (Fig. 3b). These data indicate that MHCY genes are located in discrete foci along GGA16. This finding is consistent with our inability to assemble MHCY sequence into a single coherent contig. To fully understand arrangement of genes in the RJF MHCY haplotype will require further work to identify the nature of the sequences intervening between the foci of MHCY genes and to fully assemble sequence for the region.

### Genes within MHCY

Sequence analysis revealed MHCY to be a region densely filled with genes. A total of 107 loci were located within the 639 kb representing the RJF MHCY haplotype (Table 2). As appears typical of avian genomes, the MHCY gene density (107/0.639 Mb; 167) is high and similar to the gene density found in MHCB (46/0.242 Mb; 190). The gene density in both these regions in chickens is more than double the density in human HLA (253/3.78 Mb; 67), which is one of the most gene-dense regions in the human genome (Shiina *et al.* 2007, 2009). Mapped to MHCY are members of 6 gene families (Fig. 1a). The names of the genes and the symbols assigned were made in conjunction with the Chicken Genome Nomenclature Committee. These are: (1) MHCY region, class I heavy chain (MHCY), (2) c-type lectin-like, MHCY region (YLEC), (3) MHCY region, class II beta chain (MHCY2B), (4) leukocyte receptor cluster member 9-like, MHCY region (LENG9L), (5) zinc finger protein, MHCY region (ZNFY), and (6) only zinc finger-like, MHCY region (OZFL). A full list of genes is provided in Supplementary Table 1.

**Table 2.** Loci identified in the RJF reference genome MHCY haplotype.

Gene family	Contig				Total
	1	2	3	4	
MHCY class I	20 (1P)	13 (1P)	7 (0P)	5 (2 P)	45 (4P)
YLEC	19 (12P)	10 (4P)	9 (7P)	3 (1P)	41 (24P)
MHCY2B	4 (2P)	1 (1P)	3 (2P)	0 (0P)	8 (5P)
LENG9L	3 (2P)	2 (1P)	2 (1P)	1 (1P)	8 (5P)
ZNFY	2 (0P)	0 (0P)	2 (0P)	0 (0P)	4 (0P)
OZFL	1 (0P)	0 (0P)	0 (0P)	0 (0P)	1 (0P)
Total loci	49 (16P)	26 (7P)	23 (10P)	9 (4P)	107 (37P)

The number of pseudogenes is in parentheses.

#### MHCY class I loci

Identified are 45 MHCY class I heavy chain (MHCY) loci located at fairly regular intervals across the 4 contigs (Fig. 1a). The loci are of 3 kinds—full class I genes, gene candidates, and gene fragments (pseudogenes) (Supplementary Fig. 2). Seventeen loci predicted to have full-length class I sequences encoding molecules with the typical MHC class I gene structure are grouped into 10 types (Types a–j) based on variable amino acids. These are assembled in Category 1. Each type is variously represented by 1–4 gene copies. Evidence for expression of all 10 types is available in the form of cDNA clones and/or RNA-Seq data (Supplementary Table 2). Twenty-four loci considered to be gene candidates are grouped into 12 types (Types k–w) and assembled in Category 2. These are represented by 1–3 gene copies (Supplementary Fig. 2). The predicted genes placed in Category 2 variously contain premature stop codons, alterations at intron/exon boundaries, and indels that modify the typical MHC class I gene structure. RNA-Seq data provide evidence that some Category 2 gene types may be transcribed (Supplementary Table 2). Further work is needed to determine if these variants provide functioning proteins. Additional sequences (MHCY16P, MHCY24P, MHCY43P, and MHCY45P) are gene fragments identifying the positions of MHCY class I pseudogenes.

**Fig. 4.**
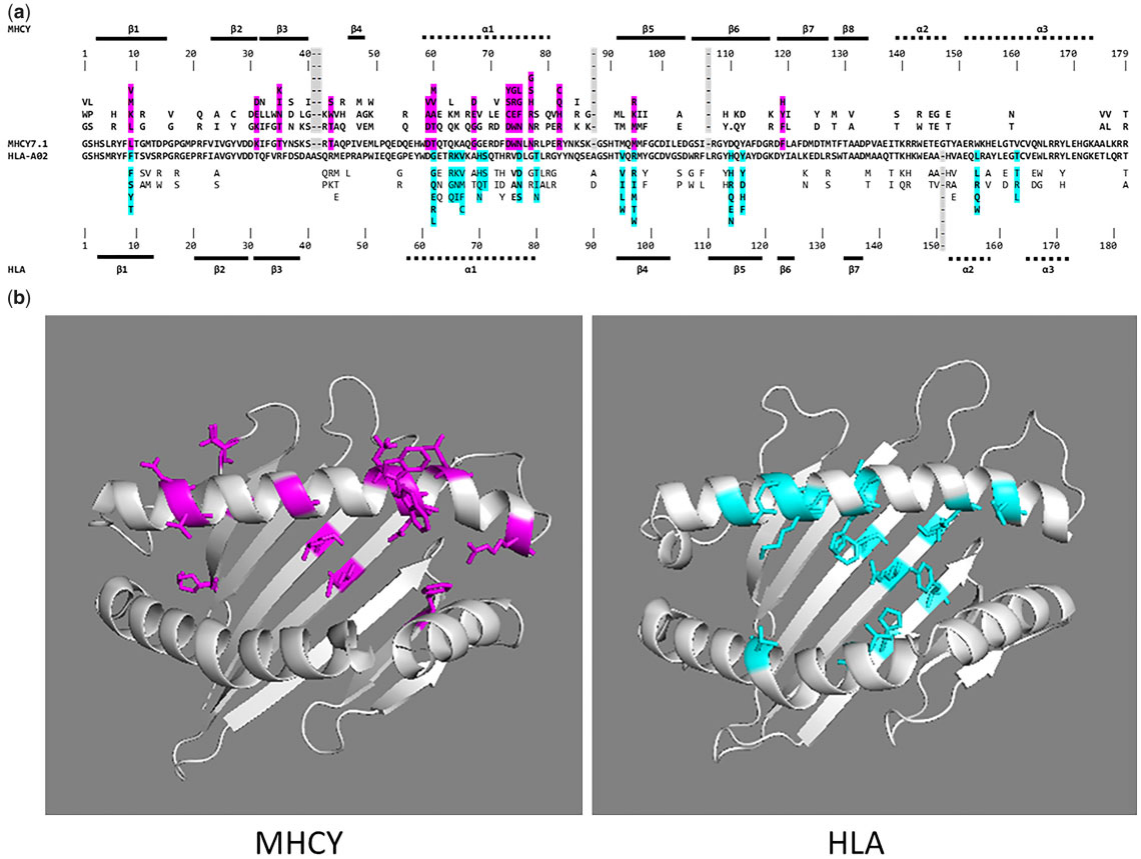
Comparison of the amino acid polymorphism present in the α1 and α2 domains of MHCY and HLA class I molecules. a) The alignment of amino acid sequences in single letter code show positions of polymorphism in the binding groove regions of MHCY and HLA class I molecules. Variability is noted for 15 MHCY class I sequences and 25 HLA sequences. For the MHCY sequences, residues with high variability defined by variability indices of 6 or greater (Supplementary Table 3) are highlighted. For HLA sequences, the highly polymorphic positions are also highlighted. Regions of β-sheets and α-helices are noted with solid and dashed bars above and below the alignments. There are 14 highly polymorphic positions in MHCY and 15 in HLA the with locations being mostly different. b) Structures of MHCY7.1 (3P73) and HLA-A02 (1HLA) with highly polymorphic positions noted. Most highly polymorphic positions in MHCY class I molecules are on the α1 helix with several other highly polymorphic positions located entirely away from the binding groove. For HLA class I, most of the polymorphic positions are located near the antigen binding groove region with many having side chains pointing into the groove.

To gain insight into MHCY class I gene polymorphism, the amino acids predicted for α1 and α2 domains were analyzed for the 10 gene types defined with full-length class I sequences. Also included were YF1*7.1 (the MHCY isoform for which there is a structure), and 4 additional cDNA clones from 2 other haplotypes (Supplementary Fig. 3). Wu/Kabat VI scores were calculated for all positions within the α1 and α2 domain sequences (Wu and Kabat 1970) (Supplementary Table 3). To be considered polymorphic, a position had to have a VI score of 6 or greater. To represent classical polymorphic MHC class I molecules in this comparison, we used 25 human HLA A and HLA B class I sequences (Supplementary Fig. S3 and Supplementary Table 3). The side chains of amino acids at most of the polymorphic positions in HLA class I molecules are in the vicinity of bound peptides within the antigen-binding groove and define peptide ligand-binding specificity (Bjorkman *et al.* 1987; Nguyen *et al.* 2021). To understand whether MHCY molecules were similar to HLA in the distribution of polymorphic positions, the α1 and α2 domain sequences predicted for MHCY and HLA were aligned (Fig. 4a). Four positions of polymorphism (MHCY 9, 60, 75, and 94) among 12 polymorphic positions in the MHCY sequences are located in positions equivalent to polymorphic positions in HLA. Two of these are in the floor of the MHCY class I ligand/antigen-binding groove (MHC 9 and 94) with side chains pointing up in the binding groove and 2 are in positions that point into the groove from the α1 helix (MHCY 60 and 75). The remaining 8 positions of polymorphism (MHCY 31, 35, 59, 67, 73, 74, 77, and 82) in MHCY differ from remainder of polymorphic residues observed in HLA (HLA 65, 66, 67, 70, 71, 80, 95, 114, 116, 156, and 163). Instead of having side chains pointing into the binding groove as occurs with most of the HLA residues, the amino acid side chains of the residues at the remaining MHCY polymorphic positions point away from the binding groove (Fig. 4b). As yet, the function of these MHCY polymorphic residues is not known. It could be that residues at these positions define specificity of MHCY class I molecular interactions with receptors. It is clear that polymorphism in MHCY class I is quite different from the polymorphic displayed by classical MHC class I molecules that bind peptide antigen. This observation together with the structural evidence showing that MHCY class I binding is too narrow to bind peptide (Hee *et al.* 2010) and the recent evidence from mass spectrometry that lysophospholipids serve as ligands for MHCY (Gugiu, Goto, Bhattacharya, Delgado, Dalton, Balendiran, and Miller, submitted), indicate that MHCY class I molecules, although polymorphic, are distinctly different from the classical MHC class I molecules in which polymorphic residues provide specificity in the binding of peptide antigens. It is not yet clear what role these distinctive polymorphic residues play in the function of MHCY class I. More studies are needed.

To begin to address the question of whether the polymorphism observed in MHCY class I might be similar in origin to the polymorphism observed at classical MHC class I loci, i.e. derived from overdominant selection (heterozygote advantage), we examined the pattern of nucleotide substitution in the codons encoding the residues within the α1 and α2 domain of MHCY molecules. Because there is not currently enough sequence data available to unequivocally define allelic sequences in different MHCY haplotypes with certainty, we used a phylogenetic tree to identify putative MHCY alleles among the sequences available in the RJF, Y5, Y7, and Y8 haplotypes. In addition, to be roughly in balance with the loci representing HLA, we selected sequences defined by 2 branches from the MHCY class I phylogenetic tree (Supplementary Fig. 9) that contain sequences from more than one haplotype. The HLA-A and HLA-B sequences identified in Supplementary Fig. 3 were used to define the HLA pattern of nucleotide substitution. We divided the sequences into 3 structurally define segments: α1 domain, α2 domain, and the remaining portions containing β-sheet and loop sequences. Values for dN and dS were determined with PAML (see GitHub for more details). In contrast to the findings for HLA where the dN/dS ratios are largely 3 or greater for both α1and α2 helices, the dN/dS ratios for the MHCY α1 and α2 domain are lower. The MHCY α1 helix, which is polymorphic, has values trending more toward neutral selection (median dN/dS = 1.24). For the MHCY α2 helix, a region lacking sequence variability, the dN/dS values are consistent with purifying selection (median dN/dS = 0.00). The remaining, nonhelix portions of the α1and α2 domains are also consistent with purifying selection (median dN/dS = 0.52). A similar value was obtained for the equivalent nonhelical portions of the HLA sequences (median dN/dS = 0.48).

#### YLEC loci

**Fig. 5.**
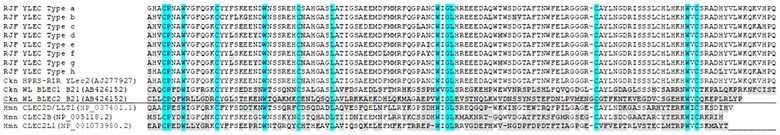
Chicken YLEC proteins are predicted to have a single C-terminal C-type lectin-like extracellular domain. The extracellular domains at the c-terminus of the predicted sequences for 8 RJF YLEC types are aligned and compared to the lectin domains encoded in chicken YLEC2, BLEC1, BLEC2 loci and 3 different human CLEC2 proteins. Positions where all 14 sequences have identical residues are highlighted. Residues that differ from the top sequence are also noted.

Forty-one c-type lectin-like loci are present within the 4 contigs. Sixteen loci appear intact and sort into 8, highly similar types (types a–h) based on their predicted amino acid sequences. The 8 types are variously represented by 1, 2, or 3 gene copies (Supplementary Fig. 4). All full-length YLEC genes are predicted to encode type II integral membrane proteins (N-terminus of the protein in the cytosol; C-terminus extracellular) bearing NK-cell domains. These full-length YLEC loci all share the same intron/exon boundary features. In contrast to the 5 exons that were predicted for YLEC loci in earlier work (Rogers *et al.* 2003), 7 exons are defined here guided by expression data in Chickspress (McCarthy *et al.* 2019) (Supplementary Fig. 4 and Table 2). The extracellular domain predicted in the YLEC sequences is most similar to the extracellular domains of LLT1 (lectin-like transcript 1, also called CLEC2D, Ocil, and Clrb), a ligand that interacts with NK cell receptor NKRP1A (CD161) and guides the activities of NK, B, and osteoclast cells (Llibre *et al.* 2016) (Fig. 5). Conserved cysteine residues and clusters of charged amino acid residues (RRR, EED, RRR) within the N-terminal region of YLEC could be the basis for covalent and charged interactions with other proteins. More work is needed to define the structure and function of YLEC-encoded proteins. The remaining loci are gene fragments identifying pseudogenes with YLEC features.

#### MHCY2B loci

Eight class IIβ chain loci are present within the MHCY contigs. Three have full-length sequences and are nearly identical (Supplementary Fig. 5). Five MHCY2B gene fragments (pseudogenes) are identified. The MHCY2B loci appear to correspond to the B-LBIII family identified in early studies before MHCY was identified (Zoorob *et al.* 1993). STAR analysis of RJF RNA-Seq data provides weak evidence for expression of the 3 full-length *MHCY2B* sequences (Supplementary Table 2).

#### LENG9L loci

There are 8 LENG9-like loci distributed across the 4 contigs (Fig. 1, Table 2). Three (LENG9L3, LENG9L5, LENG9L7) have full-length sequences (Supplementary Fig. S6). LENG9L3 and LENG9L5 are identical. LENG9L7 differs from these by only 3 predicted amino acids. LENG9L sequences are often located near MHCY2B sequences. LENG9 is identified in many jawed vertebrates, including several species of birds. LENG9 is located in MHCY in turkey (Reed *et al.* 2011). In mammalian species, LENG9 is located within the leukocyte receptor complex (Wende *et al.* 2000). Domains apparent in the LENG9 sequences are a CCCH-type zinc finger domain and an RNA cyclic group end recognition domain. Five LENG9L pseudogene loci (LENG9L1P, LENGL2P, LENG9L4P, LENG9L6P, and LENG9L8P) contain partial sequences.

#### ZNFY loci

**Fig. 6.**
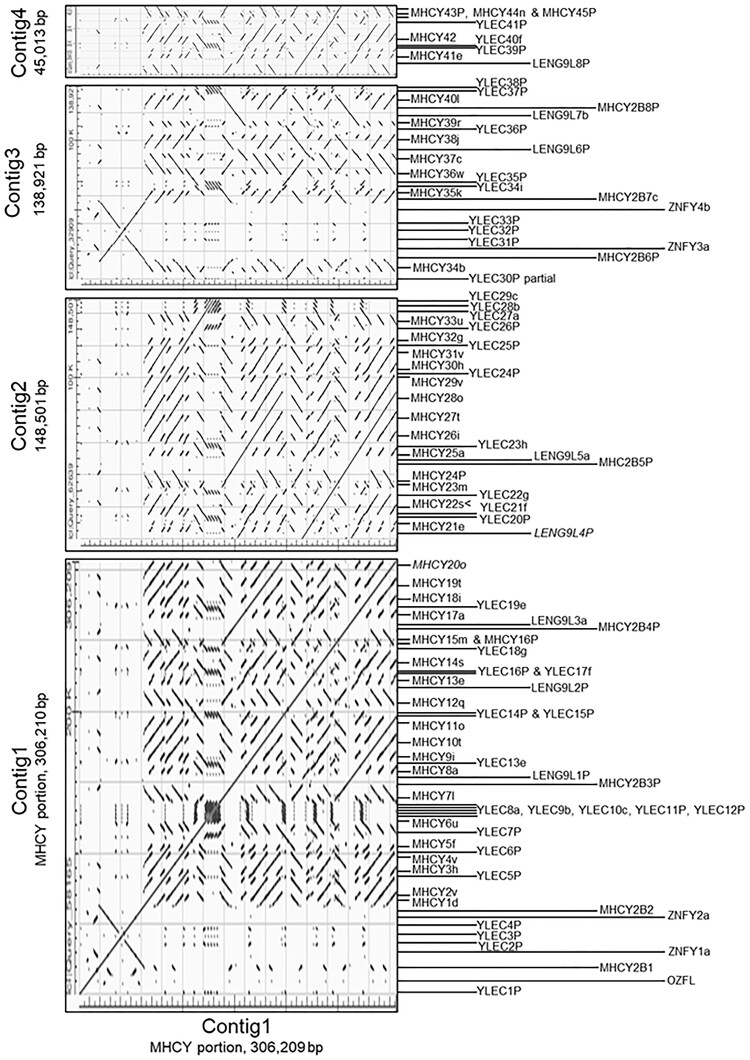
Annotated BLASTn dot plots comparing Contig 1 to itself and to the 3 other contigs provide a means for visualizing the highly repetitive nature of the RJF MHCY haplotype. The dot plots are filled with traces revealing high levels of similarity. Direct similarities are shown by traces that rise from left to right. Inverted sequence similarities are shown by traces that drop from left to right. Locations of individual genes and their orientations are noted along the side of the plots. MHCY class I loci contribute many of the traces. Matches among the clustered YLEC loci contribute series of short matching trace “patches.” Longer traces provide evidence for segmental duplications of different gene blocks. Portions of Contigs 1 and 3 containing only YLEC, MHCY2B, and ZNF genes provide evidence for a duplication resulting in an adjacent inverted repeat. Gene orientation is not directly related to the orientation of genomic segments.

There are 4 full-length ZNFY loci located within the 2 large-scale (∼40 kb) inverted sequences that are readily apparent in Contigs 1 and 3 in Fig. 6. Each gene is predicted to encode a protein with a Krüppel-associated box (KRAB) domain near the n-terminus and 8 Cys_2_-His_2_ zinc finger (ZFP) domains at the c-terminus (Supplementary Fig. 7). The KRAB domain is known to act as a transcriptional repressor domain and the ZFP domain to bind DNA (Urrutia 2003). KRAB-ZFP containing proteins are known to bind transposable elements and are thought to serve in defense against potential harm from transposition (Wolf *et al.* 2020). Currently available expression data provide weak evidence for expression of the ZNFY loci (Supplementary Table 2).

#### OZFL locus

The OZF-like gene is a single-copy gene in the RJF MHCY haplotype. OZFL is located near the margin of MHCY adjacent to the NOR (Fig. 1). The N-terminal region of OZFL defines no known domain structure. The C-terminal region contains 13 zinc finger (Cys_2_His_2_) domains (Supplementary Fig. S8). The function of OZFL is not known.

### Interspersed repetitive elements

Transposable elements are abundant in MHCY. In MHCY, the frequency is 40.4%, hence similar in frequency to interspersed repetitive elements found in the human MHC where they occupy approximately half of the sequence space. In MHCY, the repetitive elements are mostly LTR (ERVL, ERKV, and ERVL) retrotransposons elements. These fill over a quarter of the space with LINE (CR1) retrotransposons filling an additional ∼14% (Fig. 1 and Table 3). This contrasts with the chicken classical MHC (MHCB) where transposable elements are rare. In the chicken genome overall, LTR retrotransposons are estimated to be 2.3% (Mason *et al.* 2016; Warren *et al.* 2017). CR1 repeats, the most common LINE repeat in chickens, are only slightly less frequent (14% compared to 16%) in MHCY than in the genome overall.

**Table 3.** Comparison of the types and numbers of repetitive elements in MHCY and MHCB.

Repetitive sequence	MHCY^a^ (638,645 bp)			MHCB^b^ (241,833 bp)		
	Count	Total length (bp)	% total length	Count	Total length (bp)	% total length
Transposable elements						
Class I (Retrotransposons)						
** **LTR—ERV1, ERKV and ERVL	349	167,577	26.24	3	484	0.2
** **LINE—CR1	246	88,097	13.79	16	2,132	0.88
Class II (DNA transposons)						
** **hAT Charlie in MHCY; Kolobok in MHCB	2	721	0.11	1	114	0.05
Total transposable elements	597	256,395	40.14	20	2,370	1.13
Tandem repeats						
Total tandem repeats	29	7333	1.15	93	11998	4.96
Other repeats						
** **Small RNA (tRNA)	0	0	0	22	173	0.69
** **Satellites	0	0	0	1	236	0.10
** **Simple repeats	103	5,301	0.83	99	4,055	1.68
** **Low complexity	28	1,752	0.27	20	1,079	0.45
Total other repeats:	131	7,053	1.10	142	5,543	2.92

a Includes MHCY portion of Contig 1 and the full sequences of Contigs 2–4.

b AB268588.

### Duplicated gene blocks in MHCY

To gain insights into the organization of MHCY, we analyzed the RJF contigs with dot plots to visualize regions of similarity among the 4 sequences. Contig 1 was used as the base sequence against which all contigs were compared. The resulting dot plots, which are exceptionally complex, illustrate the many features shared by the 4 contigs (Fig. 6). As expected, only in the Contig 1/Contig 1 dot plot is there a line along the diagonal reflecting full identity between the compared sequences. Many off-diagonal lines in the Contig 1 to Contig 1 comparison and in comparisons of the other 3 contigs with Contig 1 show a high level of sequence similarity within and among the 4 contigs. To help define the source of these matches, the positions of the individual genes in the sequences are noted along the right margin of the plots. It is evident that a large portion of the observed patterns originate from sequence similarities shared by the many MHCY class I genes. MHCY class I genes are distributed roughly every 10 kb across most of the sequence and are variously present in forward and reverse orientations. Matches among these loci account for the off-diagonal intermediate length traces in the plots. YLEC genes also contribute to off-diagonal matches, but the dot patterns YLEC genes provide are different. The 5 closely positioned YLEC loci (YLEC8a, YLEC9b, YLEC10c, YLEC11P, and YLEC12P) in Contig 1 result distinctive patterns in the plots where YLEC gene and pseudogene sequences are present. Although not prominent, additional smaller, off-diagonal lines mark the positions of MHCY2B, LENG9L, and ZNFY genes. These patterns illustrate the high level of sequence similarity within the RJF MHCY haplotype.

Importantly, there are also longer, off diagonal lines that provide evidence for another type of sequence identity (Fig. 6). These, such as the 2 off-diagonal lines in the Contig1 to Contig 1 plot, show evidence for the duplication of segments containing multiple genes. These off-diagonal lines go beyond the shorter lines that define matches between gene family members. These appear in both forward and reverse orientations. One area of segmental duplication that is especially apparent, is near the beginning of Contig 1 where an inversion provides a distinctive pattern that is apparent again in Contig 3. In these regions, there are duplications in which a second copy is immediately adjacent to the first, but in reverse orientation. The occurrence of this pattern again in Contig3 is evidence of what may be duplication of this distinctive duplication.

**Fig. 7.**
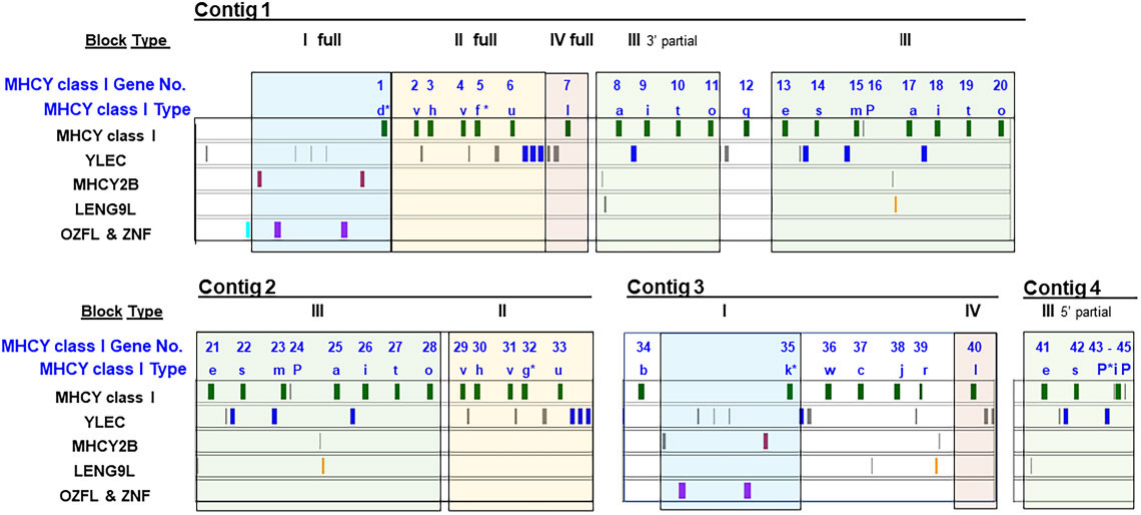
Multiple blocks of identical and near-identical sequences provide evidence that segmental duplication has contributed to the RJF MHCY haplotype. The 4 contigs contain 4 types of duplicated blocks of sequence—Block I, Block II, Block III, and Block IV. There are 2 full copies of Block I, 2 full copies of Block II, 4 copies of Block III, and 2 copies of Block IV. Full copies of Block III are present in Contig 1 and Contig 2 with additional partial copies in Contig 1 (partial 3ʹ) and Contig 4 (partial 5ʹ). An asterisk (*) has been used to identify regions of minor sequence difference found in several locations. For example, the MHCY class I genes “d” and “k” are highly similar, but not identical even though they occupy equivalent positions in different copies of Block I. Similarly, MHCY class I genes “f” and “g” are not entirely identical even though they occupy equivalent positions in different copies of Block II. The 2 copies of Block IV appear in inverted order, while the other duplicated blocks occur as direct repeats.

There are several additional longer, off-diagonal lines that identify regions of long duplicated regions. With careful analysis of dot plots and the sequences, it was possible to define 4 different duplicated gene blocks (Fig. 7). All 4 blocks are present in Contig 1 (Blocks I–IV). Second copies of Block I and Block II are present in Contig 3 and Contig 2, respectively. There is a full copy of Block III in Contig 2. Partial copies of Block III are present in Contig 1 (3ʹ partial) and Contig 4 (5ʹ partial). Block IV is a small block in Contig 1 with an inverted copy in Contig 3. Conspicuous for its lack of duplication in the sequences presently available is the region of 4 MHCY class I genes (MHCY36–MHCY39) in Contig 3. It remains to be determined whether this segment may also be duplicated. Duplicates of this region could be located in the portion of the RJF haplotype for which there is currently no sequence available.

## Discussion

This unusual gene region (MHCY) is specialized and different in many ways from the classical chicken MHCB region and classical MHC regions in mammals. Although there were early indications that MHCY was not a duplicate copy of MHCB, unanticipated at the outset of this sequencing project was how different MHCY would be in gene organization from the model provided by MHCB. Earlier phylogenetic analyses suggested MHCY class I loci are relatively young and share recent ancestry with MHCB class I (Afanassieff *et al.* 2001). With MHCY and MHCB located in the same vicinity (on the q-arm) on Chr16, it was logical at the beginning to consider them as possibly products of a relatively recent local duplication event and therefore likely to be similar in organization. MHCB has long been known as a small, compact gene region linked with other polymorphic loci within a larger haplotype (Kaufman *et al.* 1999; Hosomichi *et al.* 2008; Goto *et al.* 2009). When only 2 class I loci were identified in the first MHCY haplotype to be examined (Y7 haplotype in the inbred CB line), it continued to be reasonable to consider the possibility that MHCY would, like MHCB, have a core in which a small number of MHCY class I genes would be present and that the idea of overall similarity would bear out. Now it is clear that MHCY is distinctly different from MHCB in many features.

While MHCY and MHCB are identified by the presence of MHC class I and class II gene family members and both contain C-type lectin-like loci, the 2 regions are otherwise quite different. MHCB haplotypes are small (∼240 kb) and essentially stable in size (Hosomichi *et al.* 2008). The extended MHCB haplotype contains members of 24 gene families [BG, KIFC, BZFP, 44G24, LAO, TRIM, HEP21, tRNA, GNB2L, B-BTN, Blec, BLB (class II), TAPBP, BRD, DMA, DMB, BF (class I), TAP, C4, CenpA, CYP21, TNXB, LTB4R1, and CD1], reminiscent of the gene families that define the MHC of mammals (Shiina *et al.* 2007). Each gene family is represented either by a single locus or only a few loci. In contrast, only 6 gene families have been identified in MHCY (MHCY class I, YLEC, MHCY class IIB, LENG9L, ZNFY, and OZFL). In the RJF MHCY haplotype, there is an extraordinarily large number of MHCY class I (45) and YLEC (41) loci within the 639 kb of MHCY sequence reported here. RJF MHCY haplotype is at least 2.5 times larger than the extended MHCB haplotype.

While sequence data are needed for additional haplotypes, differences in the complexity of the restriction fragment patterns exhibited in MHCY class I Southern hybridizations indicate that the size of MHCY haplotypes may be quite variable (Fig. 2). The restriction fragment patterns for haplotypes RJF and Y4–Y6 appear complex, indicating that these are likely physically large haplotypes. In contrast, the patterns for some other haplotypes, such as Y1, Y2, Y3, and Y7, are likely far simpler, suggesting fewer genes and likely physically smaller haplotypes. Such size variation could be based on different numbers of duplicated blocks present. The 4 obvious blocks of sequence in the RJF haplotype (Fig. 7) that appear to be duplicated in part or in entirety within the RJF haplotype may be present in fewer duplications or may be entirely missing in what appear to be simpler haplotypes. There is a precedent for variation MHC haplotype size. Size variation among HLA haplotypes has been observed (Horton *et al.* 2004; Stewart *et al.* 2004). While more MHCY sequence data and investigation are needed, it could be that the numerous transposable elements present in MHCY promote recombination and contribute to the duplicated gene blocks in the RJF haplotype. A recent analysis of the extensive data available from human MHC homozygous cell lines revealed recombination sites in HLA in the vicinity of transposable elements (Kulski *et al.* 2020). It could be that transposable elements in MHCY facilitates evolution of the MHCY gene region and provides diversity that might be advantageous in populations subject to disease challenge.

Even though there is now evidence suggesting that duplicated gene blocks likely contribute to MHCY variability, much remains to be learned about the arrangement of MHCY region on GGA16. FISH hybridization of interphase nuclei of RJF revealed the presence of multiple foci of MHCY hybridization along GGA16. These results are similar to those in an earlier FISH study of an unidentified MHCY haplotype (Solinhac *et al.* 2010). Multiple MHCY foci observed with the RJF MHCY haplotype were unexpected. The basis for this gene arrangement needs further investigation. What lies in the intervals between the MHCY foci is not known. It could be that these are genes assigned to GGA16 by trisomy mapping that have not yet been localized (Miller *et al.* 2014). Among these are genes for immunoglobulin-like receptors for NK and T cells. Other genes not yet identified, such as additional c-type lectin-like genes, might be localized within those intervals. The regions might be filled with repetitive sequence. The number of foci observed in FISH could be related to the overall size of the haplotypes. It remains to be tested whether the apparently more complex haplotypes have more foci in FISH hybridization than the apparently simpler ones (Fig. 2).

This study has further defined the unusual nature of the MHC class I loci located in MHCY. Early work provided evidence that MHCY class I genes encode a structurally distinct type of polymorphic MHC class I molecule. The first indication of this was when tyrosine residues essential in the binding of peptide within the binding groove were found lacking in YFVw*7.1 (Afanassieff *et al.* 2001). A structural determination for YFVw*7.1 revealed a hydrophobic binding groove too narrow to accommodate peptide ligands (Hee *et al.* 2010). Recent mass spectrometry revealed the presence of lysophospholipids within the binding groove of YFVw*7.1 expressed as recombinant protein and refolded in the presence of a total lipid extract (Gugiu, Goto, Bhattacharya, Delgado, Dalton, Balendiran, and Miller, submitted). The sequence data in this study add further evidence for the specialized nature of the MHCY class I molecules. The unusual positions of polymorphic residues in MHCY class I molecules have become clearly evident in this study. Most of the MHC class I polymorphic amino acid residues appear in locations where the side chains point away from the binding groove. While more work is clearly needed to define the selective forces driving MHCY class I polymorphisms, the preliminary test in this study suggests MHCY polymorphism is driven by selective forces different from the over-dominant selection associated with variability in classical class I molecules. Findings are consistent with the hypothesis that the MHCY molecules are a specialized group of polymorphic MHC class I molecules distinctly different from the typical MHC class I molecules that occur in different organisms in a variety of configurations. Instead, MHCY might be thought of as an elaborate, specialized expansion of the MHC region that has occurred in chickens under conditions that remain to be defined.

How MHC class I sequence polymorphism links to immune responses is not yet clear. An appealing hypothesis is that MHCY class I molecules guide early immune responses similar to MR1, such that the level of expression guides downstream responses by specialized immune cells (Kjer-Nielsen *et al.* 2012; McWilliam and Villadangos 2017; McWilliam and Salio 2021). It may also be that the YLEC loci contribute to guiding or modulating these responses. These interesting genes and their polymorphism suggest MHCY is under selection in host/pathogens interactions and that there might be a selective advantage in the maintenance of polymorphism. Much remains to be learned about MHCY and immunity.

## Supplementary Material

jkac218_Supplementary_Figures_S1-S9

jkac218_Supplementary_Methods

jkac218_Supplementary_Tables_S1-S3

## Data Availability

All sequence data are deposited in GenBank. Details of sequence analysis, in addition to those provided here, are publicly available in GitHub: https://github.com/cwarden45/Miller_Red_Jungle_Fowl_MHCY/ Supplemental material is available at G3 online.
