# Supplementary material for: The Gallus gallus RJF reference genome reveals an MHCY haplotype organized in gene blocks that contain 107 loci including 45 specialized, polymorphic MHC class I loci, 41 C-type lectin-like loci, and other loci amid hundreds of transposable elements
Source: G3 (Bethesda). 2022 Aug 23;12(11):jkac218. doi: 10.1093/g3journal/jkac218 (PMC9635633; doi:10.1093/g3journal/jkac218)

Figure S1. **SMRT sequencing coverage for each BAC clone.**

The seven BAC clones containing MHYC sequence have extensive coverage across their lengths. Coverage is plotted here based on per base depth using a window size of 200 bp.
Please note that coverage scale varies among the plots.

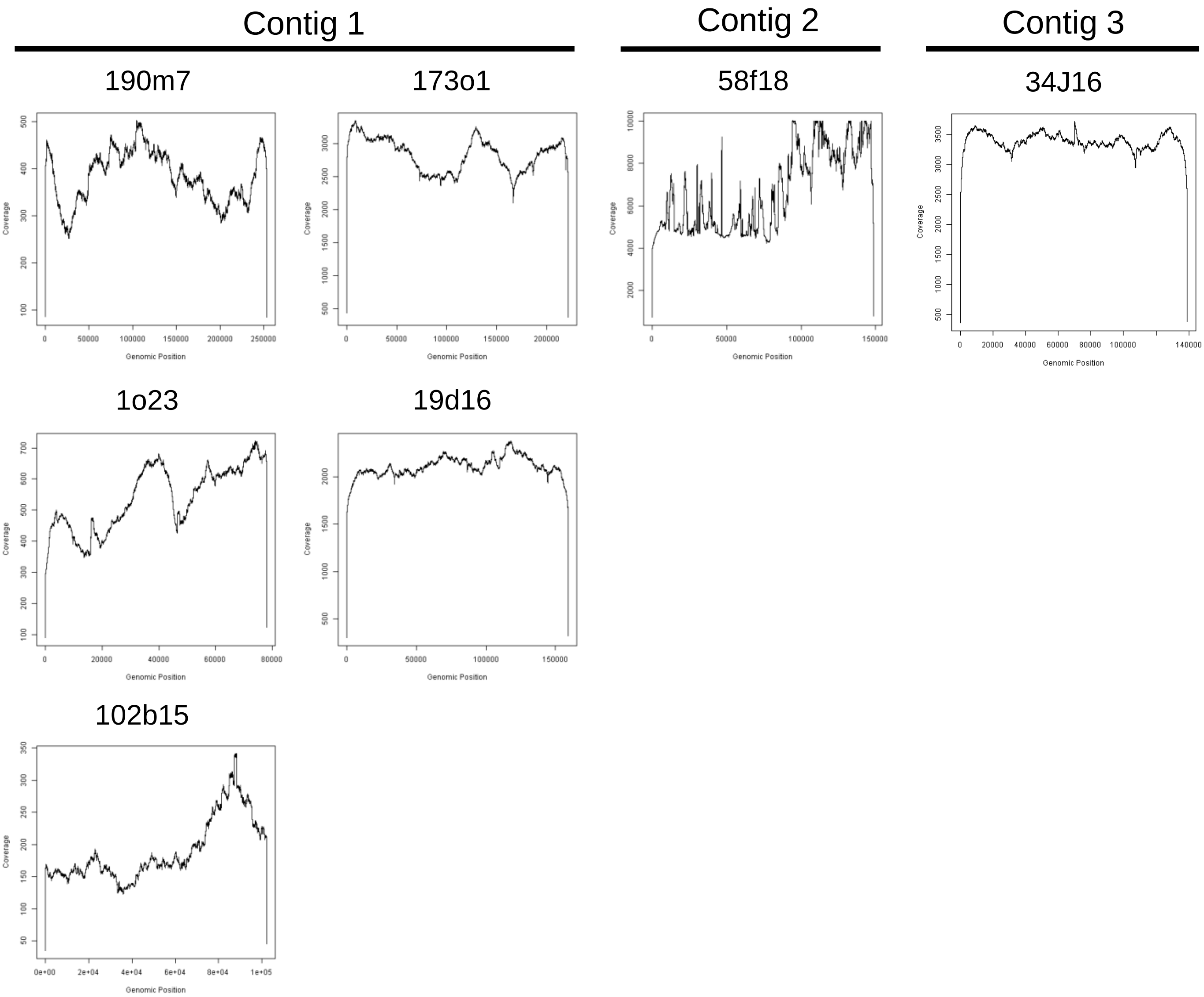

Figure S2. **Alignment of the predicted amino acid sequences for the MHCY class I loci within Contigs 1-4.**

The contigs contain 45 MHCY class I loci. Only four of these, MHCY16P, MHCY24P, MHCY43P, and MHCY45P, are gene fragments and are designated as pseudogenes. The remaining 41 loci group into 22 types in which there are variously one to four gene copies. Ten types (Types a-j) encode molecules with the typical class I structure consisting of sequence encoding signal peptide, three extracellular domains, transmembrane domain, and cytoplasmic region. These are placed in Category 1. There are cDNA clones for three of the types in Category I and RNA-Seq data supporting the expression of all ten Category 1 types (**Table S3**). The remaining 24 types, identifying candidate genes, are placed in twelve different types (Types k-w) in Category 2. In the Category 2 the sequences variously contain exon/intron boundary mutations, premature stop codons, and insertions/deletions. Expression of some Category 2 loci is supported by RNA-Seq data (**Table S3**). Residues that vary from those in first sequence listed are highlighted in grey in the alignment below.

		**Signal Peptide (exon 1)**
**Category 1 MHCY class I loci with typical MHC class I structure**		
**a)** Loci with cDNA clones and RNA-Seq data		
**Type MHCY-a**	(MHCY8, MHCY17, MHCY25)	>MGPSKVVVLGLLLGALGAEAC
**Type MHCY-b**	(MHCY34)	>MGPSEAVLLGLLLGALGAAEC
**Type MHCY-c**	(MHCY37)	>MGPSEVVVLGLLLGALGAAAC
**b)** Additional loci with RNA-Seq data		
**Type MHCY-d**	(MHCY1)	>MGPSEVVVLGLLLGALGAAVC
**Type MHCY-e**	(MHCY13, MHCY21, MHCY41)	>MGPSEVVVLGLLLGSLGAAEC
**Type MHCY-f**	(MHCY5)	>MGPSEAVVLGLLLGSLGAAVC
**Type MHCY-g**	(MHCY32)	>MGPSEAVVLGLLLGSLGAAVC
**Type MHCY-h**	(MHCY3, MHCY30)	>MGPSEVVVLGLLLGALGAAAC
**Type MHCY-i**	(MHCY9, MHCY18, MHCY26)	>MGPSEAVVLGLLLGALGAAEC
**Type MHCY-j**	(MHCY38)	>MGPCKAVLLGLLLGALGAAAC
**Category 2 MHCY class I-like candidate genes**		
**a)** Loci with exon/intron boundary mutations		
**Type MHCY-k**	(MHCY35)	>MGPSEAVLLGLLLGALGAAAC
**Type MHCY-l**	(MHCY7, MHCY40l	>MGPSEAVLLGLLLGALGAAAC
**Type MHCY-m**	(MHCY15, MHCY23)	>MGPCKAVLLGLLLGALGAAAC
**Type MHCY-n**	(MHCY44)	>MGTCKAVLLGLLRGALGAAAC
**Type MHCY-o**	(MHCY11, MHCY20, MHCY28)	>MGPCKAVLLGLLLGALGAAAC
**Type MHCY-q**	(MHCY12)	>MGPSEAVVLGLLLGALGAAEC
**b)** Loci with premature stops		
**Type MHCY-r**	(MHCY39)	>MGPSEAVLLGLLLGALGMAAC
**Type MHCY-s**	(MHCY14, MHCY22, MHCY42)	>MGPSEVVVLGLLLGALGAAAC
**Type MHCY-t**	(MHCY10, MHCY19, MHCY27)	>MAPSEAVVLGLLLGALGMVAC
**c)** Loci with insertions/deletions and additional mutations		
**Type MHCY-u**	(MHCY6, MHCY33)	>MGPSEAVVLGLLLG___AAEC
**Type MHCY-v**	(MHCY2, MHCY4, MHCY29, MHCY31)	>MGPSEVVVLGLGLGLQLLVGALGAAVC
**Type MHCY-w**	(MHCY36)	>MGPSEVVVLGLRLRLRLRLRLRLRLRLRLRLRLRLLLLLLLLLLLLLLLLLLLLGALGAAVC

Alpha 1 domain (exon 2)

Category 1 MHCY class I loci with typical MHC class I gene structure

a) Loci with cDNA clones and strong support from RNA-Seq data

Type	Loci	Sequence
**Type MHCY-a**	(MHCY8, MHCY17, MHCY25)	WPHSLRYFVTGMTDPGPGMPRFVIVGYVDGDLFGKYDSKIKSAQPIVEMLPQEDQEHWAVQTQKARGGERDFDWFLSRLPERYNKSG
**Type MHCY-b**	(MHCY34)	GSHSLRYFMTRMTDPGPGMPQFVIVGYVDGELFGKYDSKSRWVHPIVEKLPQEDREHWVAQTQKARDGELEFSEGLHRLQVRYNRSG
**Type MHCY-c**	(MHCY37)	WPHSLRYFLTGMTDPGPGMPRFVIVGYVDGDLFGKYDSKIKSAQPIVEKLPQEDQEHWDTQTQKARDGELDFCGFLGSLPEQYNKSG

b) Additional loci with cDNA clones currently lacking, but supported by RNA-Seq data

Type	Loci	Sequence
**Type MHCY-d**	(MHCY1)	GLHSLRYFLTGMTDPGPGMPRFVIVGYVDDKIFGIYDSKSRTAQPIVEMLPQEDREHWAAQTQKAQGGERDFDRGLGRLPERYNKSK
**Type MHCY-e**	(MHCY13, MHCY21, MHCY41)	VPHSLRYFLTGMTDPGPGMPRFVIVGYVDGDLFGKYDSKSRTAQPIVEMLPQEDQEHWAMQTQKARGGERDFDRGLRRLPERYNRSG
**Type MHCY-f**	(MHCY5)	GSHSLHYFLTGMTDPGPGMPQFVIVGCVDGELLWNYSSLGRTVRPIMGWLPQEDQEHWDAETKKARDVELDFYEFLGRLQVHYNKSG
**Type MHCY-g**	(MHCY32)	GSHSLHYFMTGMTDPGPGMPQFVIVGCVDGELLWNYSSLGRTVRPIMGWLPQEDQEHWDAETKKARDVELDFYEFLGRLQVHYNKSG
**Type MHCY-h**	(MHCY3, MHCY30)	GSHSLRYFKTRMTDPGPGMPQFVIVGYVDGELFGKYDSKSRWVHPIAEMLPQEDREHWDTQTQKARDVELVFCGFLGRLQVHYNKSG
**Type MHCY-i**	(MHCY9, MHCY18, MHCY26)	GPHSLRYFMTRMTDPGPGMPQFVIVGYVDGELFGKYDSKSRWVHPIVEKLPQEDREHWVAQTQKAREGELEFSEGLHRLQVRYNRSG
**Type MHCY-j**	(MHCY38)	GSHSLRYFLTGMTDPGPGMPRFVAVGYVDDKIFGKYDSKSRWVHPIVEMLPQEDQEHWDTQTQMAREGELEFSEGLHRLQVRYNRSG

Category 2 MHCY class I-like candidate genes

a) Loci with exon/intron boundary mutations

Type	Loci	Sequence
**Type MHCY-k**	(MHCY35)	GSHSLRYFKTRMTDPGPGMPQFVIVGCVDGELLWNYNSLGRTVRPIMGWLPQEDQEHWDAETQKARDVELHFYEFLGRLQVHYNKSG
**Type MHCY-l**	(MHCY7, MHCY40)	GSHSLRYFKTRMTDPGPGMPQFVIVGCVDGELLWNYNSLGRTVRPIVGWLPQEDQEHWDAETQKARDVELHFYEFLGRLQVHYNKSG
**Type MHCY-m**	(MHCY15, MHCY23)	GSHSLRYFKTRMTDPGPGMPQFVIVGYVDGELLWNYNSLCRTVRPIMGWLPQEDQEHWDAETKKARDVELDFYEFLGRLQVHYNKSG
**Type MHCY-n**	(MHCY44)	GSHSLRYFKTRMTDPGPGMPQFVIVGYVDGELLWNYNSLCRTVRPIMGWLPQEDQEHWDAETKKARDVELDFYEFLGRLQVHYNKSG
**Type MHCY-o**	(MHCY11, MHCY20, MHCY28)	GSHSLRYFLTGMTDPGPGMPRFVIVGYVDGDLFGKYDSKSRWVHPIVEKLPQEDREHWAAQTEKARGGERDFDRGLGSLPERYNKSG
**Type MHCY-q**	(MHCY12)	GSHSLRYFMTGMTDPGPGMPRFVIVGYVDGELFGKYDSKSRWVHPIVEKLPQEDREHWDGQSQKARDGELEFSEGLHRLQVRYNRSG

b) Loci with premature stops

Type	Loci	Sequence
**Type MHCY-r**	(MHCY39)	WSHSLRYFVTGMTDPGPGMPRFVIVGYVDGDLFGKYDSKIKSAQPIVEKLPQEDQEHWAAQTQKARGGERDFDWFLSRLPERYNKSK
**Type MHCY-s**	(MHCY14, MHCY22, MHCY42)	GSHSLRYFKTRMTDPGPGMPQFVIVGYVDGELFGKYDSKSRWVHPIAEMLPQEDREHWDTQTQKARDVELVFCGFLGRLQVHYNKSG
**Type MHCY-t**	(MHCY10, MHCY19, MHCY27)	GSHSLRYFKTRMTDPGPGMPQFVIVGYVDGELFGKYDSKSRWVHPIAEMLPQEDREHWDTQTQKARDVELVFCGFLGRLQVHYNKSG

c) Loci with insertions/deletions and additional mutations

Type	Loci	Sequence
**Type MHCY-u**	(MHCY6, MHCY33)	GSHSLRYFMTGMTDPGPGMPQFVIVGYVDGELFGKYDSKSRWVHPIVEKLPQEDQEHWDTQTLKAREGELEFSEGLHRLQVRYNRSG
**Type MHCY-v**	(MHCY2, MHCY4, MHCY29, MHCY31)	GSHSLRYFLTGMTDPGPGMPRFVIVGYVDDKIFGIYDSKSRTAQPIVEMLPQEDQEHWAAQTQKAQGGERDFDWFLSRLPERYNKSG
**Type MHCY-w**	(MHCY36)	GLHSLRYFLTGITNPGPGMPRFVIVGYVDDKIFGIYDSKSRTAQPIVEMLPQEDQEHWAVQTQKAQGGERDFDWFLSRLPERYNKSK

Alpha2 domain (exon 3)

Category 1 MHCY class I loci with typical MHC class I gene structure

a) Loci with cDNA clones and strong support from RNA-Seq data

**Type MHCY-a**	(MHCY8, MHCY17, MHCY25)	GSHTLQRMIGCDILADGSIRGHDKYAFDGRDYIAFDMDTMTFTAADPVAEITKRRWETEGTYAERWKHELGTVCVQNLRRYLEHGKAALKRR
**Type MHCY-b**	(MHCY34)	GSHTLQKMFGCDILEDGSIRGYDQDAFDGRDYIAFDMDTMTFTAADPVAEISKRRREEEGTYAERWKHELGTVCVQNLRRYLEHGKAALKRR
**Type MHCY-c**	(MHCY37)	GSHTLQMMFGCDILEDGSIRGYDQYAFDGRDHIAFDMDTMMFTAADPVAEITKRRWEEEGEYAERWKHELGNVCVQNLRRYLEHGKAALKRR

b) Additional loci with cDNA clones currently lacking, but supported by RNA-Seq data

**Type MHCY-d**	(MHCY1)	GSHTLQMMFGCDILEDGSIRGYDQYAFDGRDYIAFDMDTMTFTAADPVAEITKRRWETEGTYAERWKHELGTVCVQNLRRYLEHGKAALKRR
**Type MHCY-e**	(MHCY13, MHCY21, MHCY41)	GSHTLQKMFGCDILEDGSIRGYDQYAFDGRDFLAFDMDTMTFTAADPVAEITKRRWEEEGTYAERWKHELGTVCVQNLRRYLEHGKAALKRR
**Type MHCY-f**	(MHCY5)	GSHTLQKMFGCDILEDGSIRGYDQYAFDGRDFLAFDMDTMTFTAADPVAEITKRRWEEEGTYAERWKHELGTVCVQNLRRYLEHGKAALKRR
**Type MHCY-g**	(MHCY32)	GSHTLQKMFGCDILEDGSIRGYDQYAFDGRDFLAFDMDTMTFTAADPVAEITKRRWEEEGTYAERWKHELGTVCVQNLRRYLEHGKAALKRR
**Type MHCY-h**	(MHCY3, MHCY30)	GSHTLQKMFGCDILEDGSIRGYDQYAFDGRDFLAFDMDTMTFTAADPVAEITKRRWEEEGTYAERWKHELGTVCVQNLRRYLEHGKAALKRR
**Type MHCY-i**	(MHCY9, MHCY18, MHCY26)	GSHTLQKMFGCDILEDGSIRGYDQDAFDGRDYIAFDMDTMTFTVADPVAEISKRRREEEGTYAERWKHELGTVCVQNLRRYLEHGKAALKRR
**Type MHCY-j**	(MHCY38)	GSHTLQKMFGCDILEDGSIRGYDQYAFDGRDFLAFDMYTMTFTAADPVAEITKRRWETGGTYAERWKHELGTVCVQNLRRYLEHGKAALKRR

Category 2 MHCY class I-like candidate genes

a) Loci with exon/intron boundary mutations

**Type MHCY-k**	(MHCY35)	GSHTLQKMIGCDILEDGSIRGYVQYAFDGRDFLAFDMDTMTFTAADPVAEITKRRWEEEGIYTEGCKHELGTICVQNLRRYLEHGKAVLKRR
**Type MHCY-l**	(MHCY7, MHCY40)	GSHTLQKMIGCDILEDGSIRGYVQYAFDGRDFLAFDMDTMAFTAADAVAEITKRRWEEEGIYTEGCKHELGTICVQNLRRYLEHGKAALKRR
**Type MHCY-m**	(MHCY15, MHCY23)	GSHTLQKMIGCDILEDGSIRGYVQYAFDGRDFLAFDMDKMTFTAADAVAEISKRRWEEEGIYTEGCKHELGTICVQNLRRYLEHGKAVLKRR
**Type MHCY-n**	(MHCY44)	GSHTLQKMIGCDILEDGSIRGYVQYAFDGRDFLAFDMDKMTFTAADAVAEISKRRWEEEGIYTEGCKHELGTICVQNLRRYLEHGKAVLKRR
**Type MHCY-o**	(MHCY11, MHCY20, MHCY28)	GSHTLQKMFGCDILEDGSIRGYDQYAFDGRDFLAFDMDTMMFTAADPVAEITKRQWETEGTYAERWKHELGTVCVQNLRRYLEHGKAALKRR
**Type MHCY-q**	(MHCY12)	GSHTLQKMFGCDILEDGSIRGYDQDAFDGRDYIAFDMDTMTFTAADPVAEISKRRREEEGTYAERWKHELGTVCVQNLRRYLEHGKATLKRS

b) Loci with premature stops

**Type MHCY-r**	(MHCY39)	GLTRCRRCLAVTSWRTAASEGTISMHLMGGTSLPLIWTR*
**Type MHCY-s**	(MHCY14, MHCY22, MHCY42)	GSHTLQKMFGCDILEDGSIRGYDQYAFDGRDFLAFDMDTMTFTAADPVAEITKRRWEEEGTYAERWKHELGTVCVQNLRRYLEHGKAALKRR
**Type MHCY-t**	(MHCY10, MHCY19, MHCY27)	GSHTLQKMFGCDILEDGSIRGYDQYAFDGRDFLAFDMDTMTFTAADPVAEITKRRWEEEGTYAERWKHELGTVCVQNLRRYLEHGKVALKRR

c) Loci with insertions/deletions and additional mutations

**Type MHCY-u**	(MHCY6, MHCY33)	GSHTLQKMFGCDILEDGSIRGYDQYAFDGRDYIAFDMDTMTFTAADPVAEITKRRWETEGTYAERWKHELGTVCVQNLRRYLEHGKAALKRR
**Type MHCY-v**	(MHCY2, MHCY4, MHCY29, MHCY31)	GSHTMQMMIGCDILEDGSIRGYDQYAFDGRDFLAFDMDTMTFTAADPVAEITKRRWETEGTYAERWKHELGTVCVQNLRRYLEHGKAAVKRR
**Type MHCY-w**	(MHCY36)	GSHTMQMMIGCDILEDGSIRGYDQYAFDGRDFLAFDMDTMTFTAADPVAEITKRRWEEEGTYAERWKHELGTVCVQNLRRYLEHGKAAVKRR

Alpha3 domain (exon 4)

Category 1 MHCY class I loci with typical MHC class I gene structure

a) Loci with cDNA clones and strong support from RNA-Seq data

**Type MHCY-a**	(MHCY8, MHCY17, MHCY25)	ERPEVRVWGKEANGNLTLSCHAHGFYPRPIAISWMKDGMVGDQETHCGGVVPNSDGTYHASAVINVLPKDGDKYWCRVEHASLPQPSLFLW
**Type MHCY-b**	(MHCY34)	MQPEVRVWRKEADGILTLSCHAHGFYPRPIAISWIKDGMVRDQETHWGGVVPNSDGTYYASAAIDVLPEDGDKYWCRVEHASLPQPGLFLW
**Type MHCY-c**	(MHCY37)	VQPVVRVWGKEADGILTLSCHAHGFYPWPIAISWMKDGMVRDQETHWGGVVPNRDGTYHASAAIDVLPEDGDKYQCRVEHASLPQPGLFSW

b) Additional loci with cDNA clones currently lacking, but supported by RNA-Seq data

**Type MHCY-d**	(MHCY1)	VQPEVRVWRKEANGILTLSCHAYGFYPRPIAISWMKDGMVRDQETHWGGVVPNSDGTYHASAAIDVLPEDGDKYWCRVEHASLPQPGLFSW
**Type MHCY-e**	(MHCY13, MHCY21, MHCY41)	ERPKVRVWGKEADGILTLFCHAYGFYPRPIAISWMKDGMVRDQETHWGGVVPNSDGTYHASANIDVLLEDGDKYQCCVEHASLPQPGLFLW
**Type MHCY-f**	(MHCY5)	ERPKVRVWGKEADRILTLSCRAYGFYPRPIAISWMKDGMVRDQETHWGGIMPNRDGTYYASAVINVLPEDGDKYQCRVEHASLPQPGLFLW
**Type MHCY-g**	(MHCY32)	ERPKVRVWGKEADRILTLSCRAYGFYPRPIAISWMKDGMVRDQETHWGGIMPNRDGTYYASAVINVLPEDGDKYQCRVEHASLPQPGLFLW
**Type MHCY-h**	(MHCY3, MHCY30)	ERPKVRVWGKEADRILTLSCRAYGFYPRPIAISWMKDGMVRDQETHWGGIMPNRDGTYYASAVINVLPEDGDKYQCRVEHASLPQPGLFLW
**Type MHCY-i**	(MHCY9, MHCY18, MHCY26)	MQPEVRVWRKEADGILTLSCHAHGFYPRPIAISWIKDGMVRDQETHWGGVVPNSDGTYHASAAIDVLPEDVDKYWCRVEHTSLPQPGLFLW
**Type MHCY-j**	(MHCY38)	VQPEVRVWGKEADGILTLSCHAHGFYPRPISISWMKDGMVRDQETHWGGVVPNSDGTYHTSAAIDVLPEDGDKYWCRVEHASLPQPGLFSW

Category 2 MHCY class I-like candidate genes

a) Loci with exon/intron boundary mutations

**Type MHCY-k**	(MHCY35)	ERPEVRVWGKEANGILTLSCRAYGFYPRRIAISWMKDGMVRDQETHWGGIVPNSDGTYHASAAIDVLPKDVDKYWCRVEHTSLPQPGLFSW
**Type MHCY-l**	(MHCY7, MHCY40)	ERPEVRVWGKEADGILTLSCRAYGFYPRPIAISWMKDGMVRDQETHWGGMVPNSDGTYHASATIDVLPEDVDKYWCRVEHTSLPQPGLFSW
**Type MHCY-m**	(MHCY15, MHCY23)	ERPEVRVWGKEANGILTLSCRAYGFYPRPIAISWMKDGMVRDQETHWGGIVPNSDGTYHASAAIDVLPEDGDKYRCRVEHASLPQPGLFSW
**Type MHCY-n**	(MHCY44)	ERPEVRVWGKEANGILTLSCRAYGFYPRPIAISWMKDGMVRDQETHWGGIVPNSDGTYHASAAIDVLPEDGDKYRCRVEHASLPQPGLFSW
**Type MHCY-o**	(MHCY11, MHCY20, MHCY28)	ERPKVRVWGKEADGILTLSCHAHGFYPRPIAISWMKDGMVRDQETQWGGVVPNSDGTYHASAAIDVLPEDGDKYWCRVEHASLPQPGLFSW
**Type MHCY-q**	(MHCY12)	VQPEVRVWGKEADGILTLSCRAYGFYPWPISISWMKDGMVRDQETHWGGVVPNSDGTYHASAAIDVLPGDGDKYRCHVEHASLPQPGLFSW

b) Loci with premature stops

**Type MHCY-r**	(MHCY39)	
**Type MHCY-s**	(MHCY14, MHCY22, MHCY42)	ERPKVRVWGKEADRILTLSCRAYGFYPRPIAISWMKDGMVRDQETHWGGIMPNRDGTYYASAVINVLPEDGDKYQCRVEHASLPQPGLFLW
**Type MHCY-t**	(MHCY10, MHCY19, MHCY27)	ERPKVRVWGKEADGILTLSCRAYGFYPRPIAISWMKDGMVRDQETHWGGIMPNRDGTYYASAVINVLPEDGDKYQCRVEHASLPQPGLFLW

c) Loci with insertions/deletions and additional mutations

**Type MHCY-u**	(MHCY6, MHCY33)	VLPEVRVWGKEANGILTLFCRAYGFYPWPISLSWMKDGMVRDQETHWGGVVPNSDGTYHASAAIDVPPEDGDKYRCHVEHASLPQPGLFLW
**Type MHCY-v**	(MHCY2, MHCY4, MHCY29, MHCY31)	VQPEVRVWGKEADGILTLSCHAHGFYPRRIAISWMKDSMVQDQETRWGGIVPNRDGTYHTSAAIDVLPEDGDKYRCRVEHASLPQPGLFSW
**Type MHCY-w**	(MHCY36)	ERPKVRVWGKEADGILTLSCHAHGFYPRPIAISWMKDGMVRDQETRWGGIVPNRDGTYHASAAIDVLPEDRDNYRCRVEHASLPQPGLFSW

Transmembrane domain (exon 5)

Category 1 MHCY class I loci with typical MHC class I gene structure

a) Loci with cDNA clones and strong support from RNA-Seq data

**Type MHCY-a**	(MHCY8, MHCY17, MHCY25)	EPQPNLIPIVAGAVVAIVAVIAVVVGLVVWKSKS
**Type MHCY-b**	(MHCY34)	EPQPNLIPIVAGAVITIVAVIAAVVGLVVWKSKS
**Type MHCY-c**	(MHCY37)	EPQPNLIPIVAGAVVAIVAVIAAVVGLVVWKSKS

b) Additional loci with cDNA clones currently lacking, but supported by RNA-Seq data

**Type MHCY-d**	(MHCY1)	EPQPNLIPIVAVAVVAIVAVIAAVVGLVVWKSKS
**Type MHCY-e**	(MHCY13, MHCY21, MHCY41)	EPQPNLIPIVAGAVVAFVAVIAVVVGLVVWKSKS
**Type MHCY-f**	(MHCY5)	EPQPNLIPIVAGAVVAIVAVIAAVVGLVVWKSKS
**Type MHCY-g**	(MHCY32)	EPQPNLIPIVAGAVVAIVAVIAAVVGLVVWKSKS
**Type MHCY-h**	(MHCY3, MHCY30)	EPQPNLIPIVAGAVVAIVAVIAAVVGLVVWKSKS
**Type MHCY-i**	(MHCY9, MHCY18, MHCY26)	EPQPNLIPIVAGAVITIVAVIAAVIGLVVWKSKS
**Type MHCY-j**	(MHCY38)	EPQPNLIPIVAGAVVAIVAVIAAVVGLVVWKRKS

Category 2 MHCY class I-like candidate genes

a) Loci with exon/intron boundary mutations

**Type MHCY-k**	(MHCY35)	ELQPNLTPSVAGAVGAIVAVIAAVVGVVVWKSKS
**Type MHCY-l**	(MHCY7, MHCY40)	EPQPNLTPSVAGAVGAIVAVIAAVVGVVVWKSKS
**Type MHCY-m**	(MHCY15, MHCY23)	EPQPNLIPVVAGVVVTTVAVIASVIGLVVWKSKS
**Type MHCY-n**	(MHCY44)	EPQPNLIPVVAGVVVTTVAVIASVIGLVVWKSKS
**Type MHCY-o**	(MHCY11, MHCY20, MHCY28)	ELQPNLIPIVAGAVVAIVAVIAAVIGLVVWKSKS
**Type MHCY-q**	(MHCY12)	EPQPNLIPIVAGAVVTIVAVIAAVVGLVVWKSKS

b) Loci with premature stops

**Type MHCY-r**	(MHCY39)	
**Type MHCY-s**	(MHCY14, MHCY22, MHCY42)	EPQPNLIPIVAGAVVAIVAVIAAVVGLVVWKSKS
**Type MHCY-t**	(MHCY10, MHCY19, MHCY27)	EPLPNLIPIVAGAVVAIVAVIAAVVRLVVWKSKS

c) Loci with insertions/deletions and additional mutations

**Type MHCY-u**	(MHCY6, MHCY33)	EPQPNLIPSGAGAVIAIVAVIAAVVGLVVWKSKSDKGRELGVRQWLQGALDSLRSARALT*
**Type MHCY-v**	(MHCY2, MHCY4, MHCY29, MHCY31)	EPQPNLIPIEAWLVVPLVVLFVALIALLVWKFLS
**Type MHCY-w**	(MHCY36)	EPLPNLIPGGAGAVVTIVFVIAAVVGLEVWKRKS

	Cytoplasmic domain (exon 6	exon 7	exon 8)
**Category 1 MHCY class I loci with typical MHC class I gene structure**			
**a)** Loci with cDNA clones and strong support from RNA-Seq data			
**Type MHCY-a** (MHCY8, MHCY17, MHCY25)	GKEKKGYEAAP	GHDGESSISAT	GSEPSI*
**Type MHCY-b** (MHCY34)	GKEKKSYEAAP	GHDGESSGSAT	GSEPSI*
**Type MHCY-c** (MHCY37)	GKEKKGYEAAP	GRDGESSISVT	GSELSI*
**b)** Additional loci with cDNA clones currently lacking, but supported by RNA-Seq data			
**Type MHCY-d** (MHCY1)	GMEKKDYEAAP	GHDEVSSSSAT	GSELSI*
**Type MHCY-e** (MHCY13, MHCY21, MHCY41)	GKEKKGYEAAP	GHDGESSISAT	GSEPSI*
**Type MHCY-f** (MHCY5)	GKEKKGYEAAP	GHNGVSSGSAT	GSELSI*
**Type MHCY-g** (MHCY32)	GKEKKGYEAAP	GHNGVSSGSAT	GSELSI*
**Type MHCY-h** (MHCY3, MHCY30)	GKEKKGYEAAP	GHNGVSSGSAT	GSELSI*
**Type MHCY-i** (MHCY9, MHCY18, MHCY26)	GKEKKSYEAAP	GHDGESSGSAT	GSEPSI*
**Type MHCY-j** (MHCY38)	GKEKKGYEAAA	GHDEVSSGSAT	GSEPSI*
**Category 2 MHCY class I-like candidate genes**			
**a)** Loci with exon/intron boundary mutations			
**Type MHCY-k** (MHCY35)	GKEKKGYEAAA	GHDGESSV*	
**Type MHCY-l** (MHCY7, MHCY40)	GKEKKGYEAAA	GHDGESSV*	
**Type MHCY-m** (MHCY15, MHCY23)	GKEKKKSYEAAA	GHDRESSVSAMVIV*	
**Type MHCY-n** (MHCY44)	GKEKKKSYEAAA	GHDRESSVSAMVIV*	
**Type MHCY-o** (MHCY11, MHCY20, MHCY28)	GKEKKGYEAAP	GHNRESSGSATALVWNGGSGGVPAVGAVPVPCTPPLGP*	
**Type MHCY-q** (MHCY12)	GKEKKSYEAAP	GHNGECSSLATGTVWDGCSGGIPGLGTVSVLCDPRCTYSRGNTGPVVPGEPPER*	
**b)** Loci with premature stops			
**Type MHCY-r** (MHCY39r)			
**Type MHCY-s** (MHCY14, MHCY22, MHCY42)	G*		
**Type MHCY-t** (MHCY10, MHCY19, MHCY27)	G*		
**c)** Loci with insertions/deletions and additional mutations			
**Type MHCY-u** (MHCY6, MHCY33)			
**Type MHCY-v** (MHCY2, MHCY4, MHCY29, MHCY31)	G*		
**Type MHCY-w** (MHCY36)	GKEKKDYEAAA	GHDGESSGSATALVWNGGSGGVPVVGAVPVPCAPPLGPRPGQYWAQPSLENPQGGESGCGCGPT*	

Figure S3. Alignment of predicted amino acid sequences defining the polymorphic positions in the binding groove region of MHCY and HLA MHC class I molecules.
A. Alignment of the predicted α1 and α2 domain sequences for 15 MHCY class I genes with typical MHCY class I gene organization. There is evidence for expression of all fifteen sequences. **B**. Alignment of the predicted α1 and α2 domain sequences 25 HLA class I genes. Variable residues at all positions are summarized at the top of each alignment. Where the variability index (VI) is six or greater, the amino acids are highlighted in pink for MHCY and in turquoise for HLA. Calculations are in **Table S3**.

A

```
MHCY7.1           GSHSLRYFLTGMTDPGPGMPRFVIVGYVDDKIFGTYNSKSRTAQPIVEMLPQEDQEHWDTQTQKAQGGERDFDWNLNRLPERYNKSKGSHTMQMMFGCDILEDGSIRGYDQYAFDGRDFLAFDMDTMTFTAADPVAEITKRRWETEGTYAERWKHELGTVCVQNLRRYLEHGKAALKRR
                  GS   R  L G    G    R  I  Y  GKIFGT N KSRTAQ  VEM     Q   DTQ QK QGG RD DWN NR PER  K K   TM MMF     E      Y.QY    R FL    D  T  A       T   W TE T          T               AL  R
                  WP   H  K R    V    Q  A  C  DELLWN D LGKWVH  AGK     R   AAE KM REV LE CEF RS QVH  R G   ML KII     A      H KD    K YI    Y  M  V       S   R EG E          N               VV  T
                  VL      M                    DN   I S  I S R  M W         VV  L    D  V SRG H    Q  I        R                        H
                          V                         K                        M            YGL S    C
                                                                                              G

01 cDNA_MHCY*7.1  GSHSLRYFLTGMTDPGPGMPRFVIVGYVDDKIFGTYNSKSRTAQPIVEMLPQEDQEHWDTQTQKAQGGERDFDWNLNRLPERYNKSKGSHTMQMMFGCDILEDGSIRGYDQYAFDGRDFLAFDMDTMTFTAADPVAEITKRRWETEGTYAERWKHELGTVCVQNLRRYLEHGKAALKRR AF218783
02 cDNA_MHCY-a    WPHSLRYFVTGMTDPGPGMPRFVIVGYVDGDLFGKYDSKIKSAQPIVEMLPQEDQEHWAVQTQKARGGERDFDWFLSRLPERYNKSGGSHTLQRMIGCDILADGSIRGHDKYAFDGRDYIAFDMDTMTFTAADPVAEITKRRWETEGTYAERWKHELGTVCVQNLRRYLEHGKAALKRR MW423627
03 cDNA_MHCY-b    GSHSLRYFMTRMTDPGPGMPQFVIVGYVDGELFGKYDSKSRWVHPIVEKLPQEDREHWVAQTQKARDGELEFSEGLHRLQVRYNRSGGSHTLQKMFGCDILEDGSIRGYDQDAFDGRDYIAFDMDTMTFTAADPVAEISKRRREEGTYAERWKHELGTVCVQNLRRYLEHGKAALKRR MW423628
04 cDNA_MHCY-c    WPHSLRYFLTGMTDPGPGMPRFVIVGYVDGDLFGKYDSKIKSAQPIVEKLPQEDQEHWDTQTQKARDGELDFCGFLGSLPEQYNKSGGSHTLQMMFGCDILEDGSIRGYDQYAFDGRDHIAFDMDTMMFTAADPVAEITKRRWEEGEYAERWKHELGNVCVQNLRRYLEHGKAALKRR MW423629
05 Gen__MHCY-d    GLHSLRYFLTGMTDPGPGMPRFVIVGYVDDKIFGIYDSKSRTAQPIVEMLPQEDREHWAAQTQKAQGGERDFDRGLGRLPERYNKSKGSHTLQMMFGCDILEDGSIRGYDQYAFDGRDYIAFDMDTMTFTAADPVAEITKRRWETEGTYAERWKHELGTVCVQNLRRYLEHGKAALKRR in MW583603
06 Gen__MHCY-e    VPHSLRYFLTGMTDPGPGMPRFVIVGYVDGDLFGKYDSKSRTAQPIVEMLPQEDQEHWAMQTQKARGGERDFDRGLRRLPERYNRSGGSHTLQKMFGCDILEDGSIRGYDQYAFDGRDFLAFDMDTMTFTAADPVAEITKRRWEEEGTYAERWKHELGTVCVQNLRRYLEHGKAALKRR in MW583603
07 Gen__MHCY-f    GSHSLHYFLTGMTDPGPGMPQFVIVGCVDGELLWNYSSLGRTVRPIMGWLPQEDQEHWDAETKKARDVELDFYEFLGRLQVHYNKSGGSHTLQKMFGCDILEDGSIRGYDQYAFDGRDFLAFDMDTMTFTAADPVAEITKRRWEEEGTYAERWKHELGTVCVQNLRRYLEHGKAALKRR in MW583603
08 Gen__MHCY-g    GSHSLHYFMTGMTDPGPGMPQFVIVGCVDGELLWNYSSLGRTVRPIMGWLPQEDQEHWDAETKKARDVELDFYEFLGRLQVHYNKSGGSHTLQKMFGCDILEDGSIRGYDQYAFDGRDFLAFDMDTMTFTAADPVAEITKRRWEEEGTYAERWKHELGTVCVQNLRRYLEHGKAALKRR in MW583604
09 Gen__MHCY-h    GSHSLRYFKTRMTDPGPGMPQFVIVGYVDGELFGKYDSKSRWVHPIAEMLPQEDREHWDTQTQKARDVELVFCGFLGRLQVHYNKSGGSHTLQKMFGCDILEDGSIRGYDQYAFDGRDFLAFDMDTMTFTAADPVAEITKRRWEEEGTYAERWKHELGTVCVQNLRRYLEHGKAALKRR in MW583603
10 Gen__MHCY-i    GPHSLRYFMTRMTDPGPGMPQFVIVGYVDGELFGKYDSKSRWVHPIVEKLPQEDREHWVAQTQKAREGELEFSEGLHRLQVRYNRSGGSHTLQKMFGCDILEDGSIRGYDQDAFDGRDYIAFDMDTMTFTVADPVAEISKRRREEGTYAERWKHELGTVCVQNLRRYLEHGKAALKRR in MW583603
11 Gen__MHCY-j    GSHSLRYFLTGMTDPGPGMPRFVAVGYVDDKIFGKYDSKSRWVHPIVEMLPQEDQEHWDTQTQMAREGELEFSEGLHRLQVRYNRSGGSHTLQKMFGCDILEDGSIRGYDQYAFDGRDFLAFDMYTMTFTAADPVAEITKRRWETGGTYAERWKHELGTVCVQNLRRYLEHGKAALKRR in MW583605
12 cDNA_MHCY-5_1  GSHSLRYFMTGMTDPGPGMPRFVIVGYVDGKIFGIYDSKSRTAQPIAEMLPQEDQEHWATQTQKARGGERDFDWFLGRLPECYNISGGSHTLQRIIGCDILEDGSIRGYDQYAFDGKDYIAFDMDTMTFTAADPVAEITKRRWETEGTYAERWKHELGTVCVQNLRRYLEHGKAALKRT MW917156
13 cDNA_MHCY-5_2  WSHSLRYFLTGMTDPVPGMPQFVIVGYVDGDLFGKYDSKSRSAQPIVEKLPQEDQEHWDTETQKARDGELDFCGFLGRLPEQYNKSGGSHTMQMMIGCDILEDGSIRGYDQYAFDGRDFLAFDMDTMTFTAADPVAEITKRRWETEGTYAERWKHELGTVCVQNLRRYLEHGKAAVKRR MW917157
14 cDNA_MHCY-8_1  GSHSLRYFLTGMTDPGPGMPRFVIVGYVDDKNFGIYDSKSRTAQPIVEMLPQEDREHWAAQTQKARDGELEFCGLLGRLPEQYNKSGGSHMLQMMFGCDILEDGSIRGYDQYAFDGRDHIAFDMDTMTFTAADPVAEITKRRWETEGTYAERWKHELGTVCVQNLRRYLEHGKAALKRR MW917158
15 cDNA_MHCY-8_2  GSHSLRYFLTGMTDPGPGMPRFVIVGYVDDKIFGTYNSKSRTAQPIVEMLPQEDQEHWDTQTQKAQGGERDFDWNLNRLPERYNKSKGSHTMQMMFGCDILEDGSIRGYDQYAFDGRDFLAFDMDTMTFTAADPVAEITKRRWETEGTYAERWKHELGTVCVQNLRRYLEHGKAALKRR MW917159
                                                                                                                     11111111111111111111111111111111111111111111111111111111111111111111111111111111
                  0000000001111111111222222222233333333334444444444555555555566666666667777777777888888888899999999990000000000111111111122222222223333333333444444444455555555556666666666777777777
                  1234567890123456789012345678901234567890123456789012345678901234567890123456789012345678901234567890123456789012345678901234567890123456789012345678901234567890123456789012345678901234567890
```

B

```
HLA-A02           GSHSMRYFFTSVSRPGRGEPRFIAVGYVDDTQFVRFDSDAASQRMEPRAPWIEQEGPEYWDGETRKVKAHSQTHRVDLGTLRGYYNQSEAGSHTVQRMYGCDVGSDWRFLRGYHQYAYDGKDYIALKEDLRSWTAADMAAQTTKHKWEAAHVAEQLRAYLEGTCVEWLRRYLENGKETLQRT
                          F SV    R      A     D Q        A QKME         G     GE RKV AHS  H VD GTLRG      A   TV R Y  DV S W F   YH Y   G      K   R      M   I KH   AAHV   L A  E T  EW   Y     E  Q T
                          S AM    S      S     G L        T P TA         R     QN GNM TQT  D AN RIALR      D   II I F  HL P G L   HR D   S      N   S      T     QR   TVRA   R V  D R  DG   H     D  E A
                          Y              T                    E                EQ QIF  NA  Y ES  N  H           L M S              Q H                            L      E   Q T    E
                          T                                                    R    S  S                        W T                E S                                   W   W      L
                          D                                                    L    C  K                          S                N F                                       D
                          H                                                         Y                             W                D L
                                                                                                                  N

01 HLA-A02:01     GSHSMRYFFTSVSRPGRGEPRFIAVGYVDDTQFVRFDSDAASQRMEPRAPWIEQEGPEYWDGETRKVKAHSQTHRVDLGTLRGYYNQSEAGSHTVQRMYGCDVGSDWRFLRGYHQYAYDGKDYIALKEDLRSWTAADMAAQTTKHKWEAAHVAEQLRAYLEGTCVEWLRRYLENGKETLQRT AJ555412
02 HLA-A01:01     GSHSMRYFFTSVSRPGRGEPRFIAVGYVDDTQFVRFDSDAASQKMEPRAPWIEQEGPEYWDQETRNMKAHSQTDRANLGTLRGYYNQSEDGSHTIQIMYGCDVGPDGRFLRGYRQDAYDGKDYIALNEDLRSWTAADMAAQITKRKWEAVHAAEQRRVYLEGRCVDGLRRYLENGKETLQRT AJ278305
03 HLA-A03:02     GSHSMRYFFTSVSRPGRGEPRFIAVGYVDDTQFVRFDSDAASQRMEPRAPWIEQEGPEYWDQETRNVKAQSQTDRVDLGTLRGYYNQSEAGSHTIQIMYGCDVGSDGRFLRGYRQDAYDGKDYIALNEDLRSWTAADMAAQITKRKWEAAHEAEQLRAYLDGTCVEWLRRYLENGKETLQRT HG794390
04 HLA-A11:01     GSHSMRYFYTSVSRPGRGEPRFIAVGYVDDTQFVRFDSDAASQRMEPRAPWIEQEGPEYWDQETRNVKAQSQTDRVDLGTLRGYYNQSEDGSHTIQIMYGCDVGPDGRFLRGYRQDAYDGKDYIALNEDLRSWTAADMAAQITKRKWEAAHAAEQQRAYLEGRCVEWLRRYLENGKETLQRT HG794391
05 HLA-A23:01     GSHSMRYFSTSVSRPGRGEPRFIAVGYVDDTQFVRFDSDAASQRMEPRAPWIEQEGPEYWDEETGKVKAHSQTDRENLRIALRYYNQSEAGSHTLQMMFGCDVGSDGRFLRGYHQYAYDGKDYIALKEDLRSWTAADMAAQITQRKWEAARVAEQLRAYLEGTCVDGLRRYLENGKETLQRT HG794379
06 HLA-A24:02     GSHSMRYFSTSVSRPGRGEPRFIAVGYVDDTQFVRFDSDAASQRMEPRAPWIEQEGPEYWDEETGKVKAHSQTDRENLRIALRYYNQSEAGSHTLQMMFGCDVGSDGRFLRGYHQYAYDGKDYIALKEDLRSWTAADMAAQITKRKWEAAHVAEQQRAYLEGTCVDGLRRYLENGKETLQRT HG794380
07 HLA-A26:01     GSHSMRYFYTSVSRPGRGEPRFIAVGYVDDTQFVRFDSDAASQRMEPRAPWIEQEGPEYWDRNTRNVKAHSQTDRANLGTLRGYYNQSEDGSHTIQRMYGCDVGPDGRFLRGYQQDAYDGKDYIALNEDLRSWTAADMAAQITQRKWETAHEAEQWRAYLEGRCVEWLRRYLENGKETLQRT HG794395
08 HLA-A29.01     GSHSMRYFTTSVSRPGRGEPRFIAVGYVDDTQFVRFDSDAASQRMEPRAPWIEQEGPEYWDLQTRNVKAQSQTDRANLGTLRGYYNQSEAGSHTIQMMYGCHVGSDGRFLRGYRQDAYDGKDYIALNEDLRSWTAADMAAQITQRKWEAARVAEQLRAYLEGTCVEWLRRYLENGKETLQRT LN999852
09 HLA-A30.01     GSHSMRYFSTSVSRPGSGEPRFIAVGYVDDTQFVRFDSDAASQRMEPRAPWIEQERPEYWDQETRNVKAQSQTDRVDLGTLRGYYNQSEAGSHTIQIMYGCDVGSDGRFLRGYEQHAYDGKDYIALNEDLRSWTAADMAAQITQRKWEAARWAEQLRAYLEGTCVEWLRRYLENGKETLQRT EU445478
10 HLA-A31:01     GSHSMRYFTTSVSRPGRGEPRFIAVGYVDDTQFVRFDSDAASQRMEPRAPWIEQERPEYWDQETRNVKAHSQIDRVDLGTLRGYYNQSEAGSHTIQMMYGCDVGSDGRFLRGYQQDAYDGKDYIALNEDLRSWTAADMAAQITQRKWEAARVAEQLRAYLEGTCVEWLRRYLENGKETLQRT M84375
11-HLA-A32:01     GSHSMRYFFTSVSRPGRGEPRFIAVGYVDDTQFVRFDSDAASQRMEPRAPWIEQEGPEYWDQETRNVKAHSQTDRESLRIALRYYNQSEAGSHTIQMMYGCDVGPDGRLLRGYQQDAYDGKDYIALNEDLRSWTAADMAAQITQRKWEAARVAEQLRAYLEGTCVEWLRRYLENGKETLQRT HG794386
12 HLA-A74:01     GSHSMRYFFTSVSRPGRGEPRFIAVGYVDDTQFVRFDSDAASQRMEPRAPWIEQEGPEYWDQETRNVKAHSQTDRVDLGTLRGYYNQSEAGSHTIQMMYGCDVGPDGRLLRGYQQDAYDGKDYIALNEDLRSWTAADMAAQITQRKWEAARVAEQLRAYLEGTCVEWLRRYLENGKETLQRT HG965151
13 HLA-B08:01     GSHSMRYFDTAMSRPGRGEPRFISVGYVDDTQFVRFDSDAASPREEPRAPWIEQEGPEYWDRNTQIFKTNTQTDRESLRNLRGYYNQSEAGSHTLQSMYGCDVGPDGRLLRGHNQYAYDGKDYIALNEDLRSWTAADTAAQITQRKWEAARVAEQDRAYLEGTCVEWLRRYLENGKDTLERA AJ295294
14 HLA-B13:01     GSHSMRYFYTAMSRPGRGEPRFITVGYVDDTQFVRFDSDATSPRMAPRAPWIEQEGPEYWDRETQISKTNTQTYRENLRTALRYYNQSEAGSHIIQRMYGCDLGPDGRLLRGHNQLAYDGKDYIALNEDLSSWTAADTAAQITQLKWEAARVAEQLRAYLEGECVEWLRRYLENGKETLQRA HM484300
15 HLA-B14:01     GSHSMRYFYTSVSRPGRGEPRFISVGYVDDTQFVRFDSDAASPREEPRAPWIEQEGPEYWDRNTQICKTNTQTDRESLRNLRGYYNQSEAGSHTLQWMYGCDVGPDGRLLRGYNQFAYDGKDYIALNEDLSSWTAADTAAQITQRKWEAAREAEQLRAYLEGTCVEWLRRHLENGKETLQRA HG794368
16 HLA-B15:01     GSHSMRYFYTAMSRPGRGEPRFIAVGYVDDTQFVRFDSDAASPRMAPRAPWIEQEGPEYWDRETQISKTNTQTYRESLRNLRGYYNQSEAGSHTLQRMYGCDVGPDGRLLRGHDQSAYDGKDYIALNEDLSSWTAADTAAQITQRKWEAAREAEQWRAYLEGLCVEWLRRYLENGKETLQRA HG794370
17 HLA-B18:01     GSHSMRYFHTSVSRPGRGEPRFISVGYVDGTQFVRFDSDAASPRTEPRAPWIEQEGPEYWDRNTQISKTNTQTYRESLRNLRGYYNQSEAGSHTLQRMYGCDVGPDGRLLRGHDQSAYDGKDYIALNEDLSSWTAADTAAQITQRKWEAARVAEQLRAYLEGTCVEWLRRHLENGKETLQRA KU319250
18 HLA-B27.02     GSHSMRYFHTSVSRPGRGEPRFITVGYVDDTLFVRFDSDAASPREEPRAPWIEQEGPEYWDRETQICKAKAQTDRENLRIALRYYNQSEAGSHTLQNMYGCDVGPDGRLLRGYHQDAYDGKDYIALNEDLSSWTAADTAAQITQRKWEAARVAEQLRAYLEGECVEWLRRYLENGKETLQRA LN877310
19 HLA-B35:01     GSHSMRYFYTAMSRPGRGEPRFIAVGYVDDTQFVRFDSDAASPRTEPRAPWIEQEGPEYWDRNTQIFKTNTQTYRESLRNLRGYYNQSEAGSHIIQRMYGCDLGPDGRLLRGHDQSAYDGKDYIALNEDLSSWTAADTAAQITQRKWEAARVAEQLRAYLEGLCVEWLRRYLENGKETLQRA AJ420239
20 HLA-B37:01     GSHSMRYFHTSVSRPGRGEPRFISVGYVDDTQFVRFDSDAASPRTEPRAPWIEQEGPEYWDRETQISKTNTQTYREDLRTLLRYYNQSEAGSHTIQRMSGCDVGPDGRLLRGYNQFAYDGKDYIALNEDLSSWTAADTAAQITQRKWEAARVAEQDRAYLEGTCVEWLRRYLENGKETLQRA AJ310358
21 HLA-B38:01     GSHSMRYFYTSVSRPGRGEPRFISVGYVDDTQFVRFDSDAASPREEPRAPWIEQEGPEYWDRNTQICKTNTQTYRENLRIALRYYNQSEAGSHTLQRMYGCDVGPDGRLLRGHNQFAYDGKDYIALNEDLSSWTAADTAAQITQRKWEAARVAEQLRTYLEGTCVEWLRRYLENGKETLQRA L36591.1
22 HLA-B39:05     GSHSMRYFYTSVSRPGRGEPRFISVGYVDDTQFVRFDSDAASPREEPRAPWIEQEGPEYWDRNTQICKTNTQTYRESLRNLRGYYNQSEAGSHTLQRMYGCDVGPDGRLLRGHNQFAYDGKDYIALNEDLSSWTAADTAAQITQRKWEAARVAEQLRTYLEGTCVEWLRRYLENGKETLQRA L36318.1
23 HLA-B51:02     GSHSMRYFYTAMSRPGRGEPRFIAVGYVDDTQFVRFDSDAASPRTEPRAPWIEQEGPEYWDRNTQIFKTNTQTYRENLRIALRYYNQSEAGSHTWQTMYGCDVGPDGRLLRGHNQYAYDGKDYIALNEDLSSWTAADTAAQITQRKWEAAREAEQLRAYLEGLCVEWLRRYLENGKETLQRA M68964.1
24 HLA-B56:03     GSHSMRYFYTAMSRPGRGEPRFIAVGYVDDTQFVRFDSDAASPREEPRAPWIEQEGPEYWDRNTQIYKAQAQTDRESLRNLRGYYNQSEAGSHTLQRMYGCDVGPDGRLLRGHDQSAYDGKDYIALNEDLSSWTAADTAAQITQRKWEAAREAEQWRAYLEGLCVEWLRRYLENGKETLQRA D85762.1
25 HLA-B58:01     GSHSMRYFYTAMSRPGRGEPRFIAVGYVDDTQFVRFDSDAASPRTEPRAPWIEQEGPEYWDGETRNMKASAQTYRENLRIALRYYNQSEAGSHIIQRMYGCDLGPDGRLLRGHDQSAYDGKDYIALNEDLSSWTAADTAAQITQRKWEAAREAEQLRAYLEGLCVEWLRRYLENGKETLQRA AJ420241
                                                                                                                     11111111111111111111111111111111111111111111111111111111111111111111111111111111111
                  00000000011111111112222222222333333333344444444445555555555666666666677777777778888888888999999999900000000001111111111222222222233333333334444444444555555555566666666667777777777888
                  12345678901234567890123456789012345678901234567890123456789012345678901234567890123456789012345678901234567890123456789012345678901234567890123456789012345678901234567890123456789012
```

Figure S4. **Predicted amino acid sequences for YLEC loci found in Contigs 1-4.**

There are 41 YLEC loci are located within the four contigs. Sixteen loci appear to be full-length intact genes of eight different types (a-h). The types are represented by one, two or three gene copies. Predicted amino acid sequences are aligned below. Exons 1, 2, 3 and a portion of exon 4 correspond to what is considered the intracellular portions of YLEC, the remaining portion of exon 4 corresponds to the transmembrane domain. Exons 5-7 correspond to the extracellular c-type lectin-like (CTLD) domain. Residues not matching those of the first sequence are highlighted in grey. Potential glycosylation sites are underlined. Cysteine residues predicted to form disulfide bonds in the folded CTLD are shown in white on black and paired residues are marked with matching letters. Additional cysteine residues, noted with ‖, in the stalk and an intracellular region could serve in dimerization. Clusters of charged residues (noted with ***) could serve in intermolecular interactions. Not shown are the sequences for 24 YLEC pseudogenes and for YLEC30 (a partial sequence at the 5'-margin of Contig2).

```
Intracellular region                                Exon 1                 Exon 2                                                Exon 3
Intron phase                                          0                                            2                                                              1
                                                     -------------------------------------Intracellular----------------------------------------
                                                                                                ***            ***
Type a (YLEC8, YLEC27)                               M| QSVQIPILKHKVSTPAEGERLNHFSEHKAAPEPPAHQRRRIPTS| SDPQLPEEDVQILLLESNTSTPAMGEGDQQETFSEHQAAPEPLGQSGGH-QW|
Type b (YLEC9, YLEC28)                               M| QSVQIPILKHKVSTPAEGERLNHFSEHKAAPEPPAHQRRRIPTS| SDPQLPEEDVQILLLESNTSTPAMGEGDQQETFSEHQAAPEPLGQSGGH-QW|
Type c (YLEC10, YLEC29)                              M| QSVQIPILKHKVSTPAEGERLNHFSEHKAAPEPPAHQRRRIPTS| SDPQLPEEDVQILLLESNTSTPAMGEGDQQETFSEHQAALEPLGQSGGH-QW|
Type d (YLEC13, YLEC19)                              M| QSVQIPVLKHKVSTPAEGERLNHFSEHKAAPEPPAHQRRRIPTS| SDPQLPEEDVHISELESNTSMPPVGEGDQRETFSEHQAAPEPPEESGEGPQW|
Type e (YLEC17, YLEC21, YLEC40)                      M| QSVQIPILKHKVSTPAEGERLNHFSEHKAAPEPPAHQRRRIPTG| SDPQLPEEDVHISVLESNTSTPAMG-GDQQETFLEHQAAPEPLGQSGEKTQW|
Type f (YLEC18, YLEC22, YLEC41)                      M| QSVQIPILKHKVSTPAEGERLNHFSEHKAAPEPPAHQRRRIPTS| SDPQLPEEDVQILLLESNTSTPTIGEGDQQETFSEHKAVTEPLGQSTEGDQW|
Type g (YLEC23)                                      M| QSVQIPVLKHKVSTPAEGERLNHFSEHKAAPEPPAHQRRRIPTS| SDPQLPEEDVHISELESNTSMPPVGEGDQRETFSEHQAAPEPPEESGEGPQW|
Type h (YLEC34)                                      M| QSVQTPILKHKVSTPAEGERLNHFSEHKAAPEPPAHQRRRIPTS| SDPQLPEEDVQILLLESNTSTPAMGEGDQQETFSEHQAALEPLGQSGGH-QW|

                                                                      Exon 4                                                    Exon 5
Intron phase                                                                              1                                                                  0
                                                     Intracell cont.  ---------TM-----------   ----Stalk----  --------------------CTLD---------------------
                                                        ‖     ***                              ‖                    a            a                   b
Type a (YLEC8, YLEC27)                               GSWCHGMGRRRSRVQLIAVHAALGALILMLVVLVIST| VCRRAPIPPFSAF GHACPNAWVGFQGKCYYFSKEENDWNSSREHCSAHGASLATIGSAEEM|
Type b (YLEC9, YLEC28)                               GSSCHGMGRRTSRVQLIAVHAALGALILMLVVLVIST| VCWRAPIPPFPGF AHVCPNAWVGFQGKCCYFLKGENDWNSSREHCNAHGASLATIGSAEEM|
Type c (YLEC10, YLEC29)                              GSWCHGMGRRRSRVQLTAVHAALGALILLLVVLVIST| VCRRAPIPPFSAF GHACPNAWVGFQGKCYYFSKEENDWNSSREHCNAHGASLATIGSAEEM|
Type d (YLEC13, YLEC19)                              GSWCHGAGRRRSRVQLIAACAALGALILVLV--VIST| VCRQVPVPPFPDF AHVCPNAWVGFQGKCYYLSKEEYDWNSSREHCNAHGASLATIGSAEEM|
Type e (YLEC17, YLEC21, YLEC40)                      GSWCHGAGRRRSRVQLIAACAALGALILVLV--VIST| VCRQVPVPPFPDF AHVCPNAWVGFQGKCYYFSEEENDWNSSREHCNAHGASLATIGSAEEM|
Type f (YLEC18, YLEC22, YLEC41)                      GSWCHGTGRRRSRVQLIAVCAALGALILMLV--VIST| VCRQVPVPSFPDF AHVCPNAWVGFQGKCYYLSKEENDWNSSREHCNAHGASLATIGSAEEM|
Type g (YLEC23)                                      GSWCHGAGRRRSRVQLIAACAALGALILVLV--VIST| VCRQVPVPPFPDF AHVCPNAWVGFQGKCYYLSKEEYDWNSSREHCNAHGAGLATIGSAEEM|
Type h (YLEC34)                                      GSWCHGMGRRRSRVQLTAVHAALGALILLLVVLVIST| VCRRAPIPPFSAF SHACPNAWVGFQGKCYYFSKEENDWNSSREHCSAHGASLATIGSAEEM|

Extracellular Region                                              exon 6                                        exon 7
                                                                                        2
                                                     -------------------------------------CTLD continued-------------------------------
                                                                                                        c             c        b
Type a (YLEC8, YLEC27)                               DFMMRFQGPANCWIGLHREEEDAQWTWSDGTAFTNW| FELRGGGRCAYLNGDRISSSLCHLHKHWVCSRADHYVLWKQKVHPQ*
Type b (YLEC9, YLEC28)                               DFMMRFQGPANCWIGLHREEEDALWTWSNVTAFTNW| FELRGGGRCAYLNGDRISSSLCHLHKHWVCSRADHYVLWKQKVHPQ*
Type c (YLEC10, YLEC29)                              DFMMRFQGPANCWIGLHREEEDALWTWSNVTAFTNW| FELRGGGRCAYLNGDRISSSLCHLHKHWVCSRADHYVLWKQKAHPQ*
Type d (YLEC13, YLEC19)                              DFMMRFQGPANCWIGLHREEEDAQWMWSDGTAFTNW| FELRGGGRCAYLNGDRISSSLCHLHKHWVCSRADHYVLWKQKVHPQ*
Type e (YLEC17, YLEC21, YLEC40)                      DFMMRFQGPAICWIGLHREEEDAQWTWSDGTAFTNW| FELRGGGRCAYLNGDRISSSLCHLHKHWVCSRADHYVLWKQKVHPQ*
Type f (YLEC18, YLEC22, YLEC41)                      DLMMRFQGPAYCWIGLHREEEDAQWTWSDGTAFTNW| FELRGGGRCAYLNGDRISSSLCHLHKHWVCSRADHYVLWKQKVHPQ*
Type g (YLEC23)                                      DFMMRFQGPANCWIGLHREEEDAQWMWSDGTAFTNW| FELRGGGRCAYLNGDRISSSLCHLHKHWVCSRADHYVLWKQKVHPQ*
Type h (YLEC34)                                      DFMMRFQGPANCWIGLHREEEDAQWTWSDGTAFTNW| FELRGGGRCAYLNGDRISSSLCHLHKHWVCSRADLYVLWKQKAHPQ*
```

Figure S5. **Alignment of the predicted amino acid sequences MHCY class II beta genes found within Contigs 1-4**.

Three highly similar, full-length genes are present. These differ by only two or three residues in predicted amino acid sequence (highlighted in grey). MHCY2B1 matches NM_001393722 (identified as a MHCB locus before MHCY was identified). Five MHC2B pseudogenes (MHCY2B3P, MHCY2B4P, MHCY2B5P, MHCY2B6P, MHCY2B8P) are not included in this alignment.

Locus	Signal Peptide (exon 1)
MHCY2B1	MPPSPGTHCSRAAARAAGTLPPPPAAAMGSGRVLVAGAVLVALVALGARLAAGTRPS
MHCY2B2	MPPSPGTHCSRAAARAAGTLPPPPAAAMGSGRVLVAGAVLVALVALGARLAAGTRPS
MHCY2B7	MPPSPGTHCSRAAALAAGTLPPPPAAAMGSGRVLVAGAVLVALVALGARLAAGTRPS

Locus	β1 domain (exon 2)
MHCY2B1	AFFQWSATIECHFLNGTERVRFLVRHVYNRQQYVHFDSDVGLFVADTVLGEPSAKLFNSQPDVLEKNRAAVEMLCNYNYEIVAPLTLQRR
MHCY2B2	AFFQWSATIECHFLNGTERVRFLVRHVYNRQQYVHFDSDVGLFVADTVLGEPSAKLFNSQPDVLEKNRAAVEMLCNYNYEIVAPLTLQRR
MHCY2B7	AFFQWSATIECHFLNGTERVRFLVRHVYNRQQYVHFDSDVGLFVADTVLGEPSANLFNSQPDVLEKNRAAVEMLCNYNYEIVAPLTLQRR

Locus	β2 domain (exon 3)
MHCY2B1	EPKVRISALQSGSLPQTDRLACYVTGFYPPEIEVKWFQNGQEETERVVSTDVIQNGDWTYQVLVVLETSPRHGDSYVCQVEHTSLQQPITQHW
MHCY2B2	EPKVRISALQSGSLPQTDRLACYVTGFYPPEIEVKWFQNGQEETERVVSTDVIQNGDWTYQVLVVLETSPRHGDSYVCQVEHTSLQQPITQRW
MHCY2B7	EPKVRISALQSGSLPQTDRLACYVTGFYPPEIEVKWFQNGQEETERVVSTDVIQNGDWTYQVLVVLETSPRHGDSYVCQVEHTSLQQPITQRW

Locus	Transmembrane domain (exon 4)	Cyto domain 1 (exon 5)	Cyto domain 2(exon 6)
MHCY2B1	EPPGDVSRSKLLMGVGGFVLGLVYLALGIFFFLCGKK	GQPDPTSP	GILN
MHCY2B2	EPPGDVSRSKLLMGVGGFVLGLVYLALGIFFFLCSKK	GQPDPTSP	GILN
MHCY2B7	EPPGDVSRSKLLMGVGGFVLGLVYLALGIFFFLCSKK	GQPDPTSP	GILN

Figure S6. Alignment of the predicted amino acid sequences for LENG9L genes found within RJF Contigs 1-4.

LENG9L genes are single exon genes. Three full-length LENG9L loci are present. Two (LENG9L3a and LENG9L5a) are identical. The third (LENG9L7b) differs by only three predicted amino acid residues. Five LENG9L pseudogenes (LENG9L1P, LENG9L2P, LENG9L4P, LENG9L6P, LENG9L8P) are also present. In GenBank LENG9-like genes are reported for many species of birds. The closest equivalent in the human genome is AAH15921.1 with 53% identity. Residues that differ from the first sequence are highlighted in grey. Highlighted in **pink** (cysteine) and **green** (histidine) residues that define the presence of a CCCH-type zinc finger domain. Highlighted in **turquoise** are residues predicted to define an RNA cyclic group end recognition domain.

Type	Part 1
**Type a** (LENG9L3a, LENG9L5a)	MDPDPRPGAPQSEPEPRSGLAEPDPNPGPKPRLGAAGAGPGSVEADSEPNRGPEDPDPDPDQRPGPAEAVPAPAPAPCRWFLEGRCRFGPR
**Type b** (LENG9L7b)	MDPDPRPGAPQSEPEPRSGLAEPDPNPGPKPRLGAAGAGPGSVEADSEPNRGPEDPDPDPDQQPGPAEPVPAPAPAPCRWFLEGRCRFGPR
**Type**	**Part 2**
**Type a** (LENG9L3a, LENG9L5a)	CRHPHPGQSPSAVPEPKPEPNREEPGAAGKKPPLRRAAAVVSRLRWDPRVDPEAATVEYRDRFVGVVERPLPEFFTGPLCDAGPADLAVPE
**Type b** (LENG9L7b)	CRHPHPGQSPSAVPEPKPEPNREEPGAAGKKPPLRRAAAVVSRLRWDPRVDPEAATVEYRDRFVGVVERPLPEFFTGPLCDAGPEDLAVPE
**Type**	**Part 3**
**Type a** (LENG9L3a, LENG9L5a)	HRIVRIRYRGCCVWDRENRIDRVFGSGGGMGTMREVLEELGEGCGE*
**Type b** (LENG9L7b)	HRIVRIRYRGCCVWDRENRIDRVFGSGGGMGTMREVLEELGEGCGE*

Figure S7. Predicted amino acid sequence for four zinc finger protein genes, ZNF1 – ZNF4.

The ZNF sequences have KRAB and zinc finger domains. Residues defining KRAB domains are highlighted in **turquoise**. Residues defining Cys_2-His_2 zinc finger domains are highlighted with Cys and His in **pink** and **green**, respectively. The four predicted amino acid differences are highlighted in **grey**.

KRAB domain (A box and B box)

```
                             e1         e2                                  e3
Type a (ZNF1, ZNF2, ZNF4)    MATQDPSQ   EPVSFADVAVYFSREEWALLDPAQRVLYRDVMLETYECVASL    APKPAMISLLEGGEEPWIPDVRGLEDTAGDLSP
Type b (ZNF3                 MATQDPSQ   EPVSFADVAVYFSREEWALLDPAQRVLYRDVMLETYECVASL    APKPAMISLLEGGEEPWIPDVRGLEDTAGDLSP

                             e4
Type 1 (ZNF1, ZNF2, ZNF4)    AGHGVAAAPDVLQKCDVTEGQWGIASVGEIRKAIQEDLEQGEHLKQQRGNPPGETARSPQDSSTGQKQPEGARSEEVCQEKRQNPNAECGNSLKRCSVLLNHHCVR
Type 2 (ZNF3)                AGHGVAAAPDVLQKCDVTEGQWGVASAGEIRKDIQEDLEQGEHLKQQRGNPPGETARSPQDSSTGQKQPEGARSEEVCQEKRQNPNAECGNSLKRCSVLLNHHCVR

                                    Cys2-His2 fold            Cys2-His2 fold            Cys2-His2 fold            Cys2-His2 fold
                             e4 cont.
Type 1 (ZNF1, ZNF2, ZNF4)    SVKRLYKCSDCTKSFKWKSHLTCHQRIHTDERPFKCPDCPKCFKTNSHLTGHQRIHKGEKPIKYCECGKGFKYSSILQRHQRIHTGERPFKCPECPKSFKNSFHLTCHQRIH
Type 2 (ZNF3)                SVKRLYKCSDCTKSFKWKSHLTCHQRIHTDERPFKCPDCPKCFKTNSHLTGHQRIHKGEKPIKYCECGKGFKYSSILQRHQRIHTGERPFKCPECPKSFKNSFHLTCHQRIH

                                    Cys2-His2 fold            Cys2-His2 fold            Cys2-His2 fold            Cys2-His2 fold
                             e4 cont.
Type 1 (ZNF1, ZNF2, ZNF4)    TGERPFKCCECGKHFKYSFVLKRHQRMHTAVGPFKCSECGKGFKFGYELQHHNRIHTEKRPFKCPECSKSFKSKSQLTYHQRTHTGERPFKCRECEMSFKYSFVLKRHQRIH
Type 2 (ZNF3)                TGERPFKCCECGKHFKYSFVLKRHQRMHTAVGPFKCSECGKGFKFGYELQHHNRIHTEKRPFKCPECSKSFKSKSQLTYHQRTHTGERPFKCRECEMSFKYSFVLKRHQRIH

                             e4 cont.
Type 1 (ZNF1, ZNF2, ZNF4)    TGERSIKCVARVYGVLRPGSVTDGAGGPTDPRPRKGIMGKGKRGKGKREKGRKTGLTSKQWQ*
Type 2 (ZNF3)                TGERSIKCVARVYGVLRPGSVTDGAGGPTDPRPRKGIMGKGKRGKGKREKGRKTGLTAKQWQ*
```

Figure S8. Predicted amino acid sequence for the zinc finger protein OZF-like (OZFL) locus within Contig1.

The OZFL gene has a single coding exon containing n-terminal region that has no defined motif and a c-terminal region that encodes thirteen tandem zinc finger (Cys_2-His_2) domains. The Cys_2-His_2 domains are aligned. Cys and His residues are highlighted in **magenta** and **green**, respectively.

N-terminal sequence with no defined motifs

```
MTTTEEVLQKCDVTEWQWGSASVGKARRDVPGGLEQGEHLKKHQGNPPGETVRNPLDFSTGQKQPEDARSKEVCQEERQNPSDECANSLKRNSGLVNCQRTHIADI
```

C-terminal C2-H2 fold domain motifs

```
RFKCLECEKRNVKSCNLLMRQQDHTGER
PFKCPECGKSFRSSSNLLRHQRIHTGER
PFKCSECGKSFRSSSDLIVHHRIHTGER
PFKCPECAKSFKSNSHLTSHQRIHTGER
PFKCQECEKSFKCSSHLIHHQHVHTGER
PFKCPECGKSFSSSSNLSDHQRIHTGER
RFKCPECGKRFSSSSNVIIHQRIHTGER
PFKCPECGKSFKSSSTLINHQRIHTEER
PFECSECGKSFKSSSDLIVHLRVHTGYK
PYKCPECGKSFKRSSELKCHQRIHTEER
PFECSECGKSFSSSSSFSYHQRVHTGER
PYQCCECGKSFKTSSYLIVHQRIHTGER
PYQCCECGKSFSSSSKLNSHKHIHRGDSPQ
```

Figure S9. Pattern of nucleotide substitutions in MHCY class I loci is different from the pattern of over-dominant selection present in human HLA class I loci.

A. Phylogenetic tree with major four branches constructed using 15 MHCY class I gene sequences originating from four MHCY haplotypes (RJF, Y5, Y7 and Y8). Two groups, tentatively identified as putative MHCY-a alleles and putative MHCY-d alleles, were analyzed for patterns of nucleotide substitution.

B. To simplify the analysis, the sequences were divided into three portions: (α1-helix) class I α1 helical region, (α2-helix) class I α2 helical region, and (non-helical) the remaining sequence corresponding to beta sheets and loops. The dN, dS and dN/dS ratio values provide evidence that the MHCY class I are distinctly different from HLA in the α1 and α2 helical regions represented by the HLA-A and HLA-B sequences in Figure S3.

C. Density plots for pairwise dN/dS comparisons provide another means of comparing MHCY (red line) and HLA class I (green line). The chicken α1 and α2 helices appear to be under neutral and purifying selection, respectively. This contrasts with the findings for the HLA α1 and α2 helices that appear to be under positive (diversifying selection). The remaining portions (non-helical α1 and α2) appear to be under neutral selection.

A

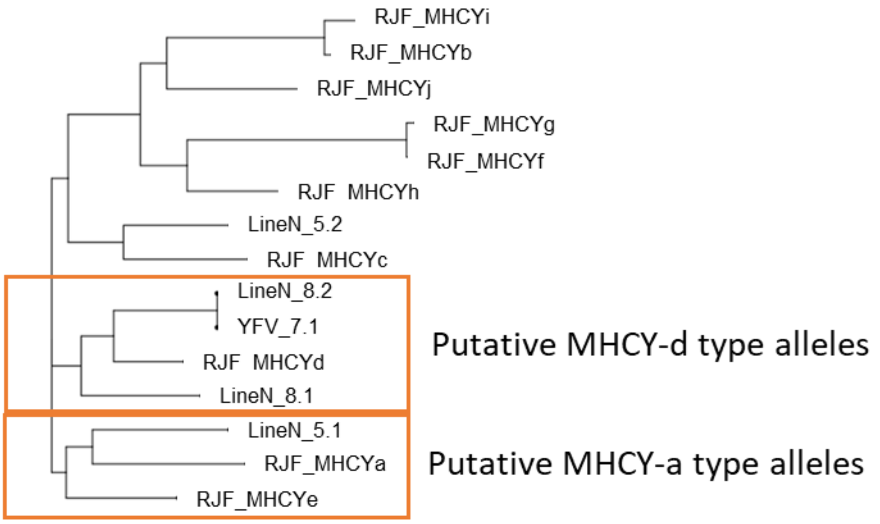

B

	dN		dS		dN/dS ratio	
	chicken	human	chicken	human	chicken	human
**α1-helix**	11.37	23.51	11.44	5.13	1.24	3.56
**α2 -helix**	0	6.39	5.14	2.4	0	3.57
**non-helical**	5.46	5.11	11.4	11.59	0.52	0.48

C

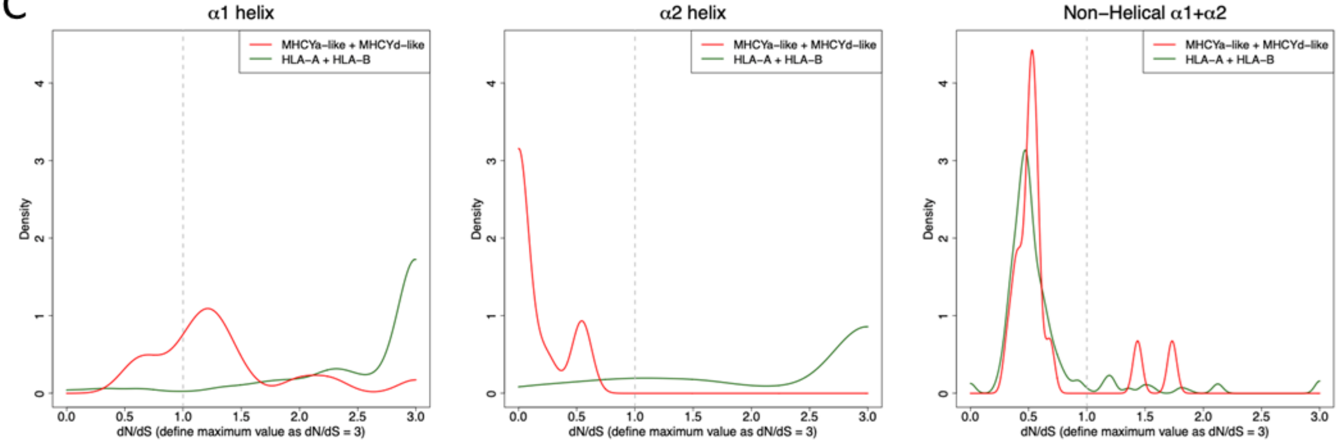

Supplement: jkac218_Supplementary_Figures_S1-S9 [file jkac218_supplementary_figures_s1-s9.pdf]
