# Supplementary material for: The Gallus gallus RJF reference genome reveals an MHCY haplotype organized in gene blocks that contain 107 loci including 45 specialized, polymorphic MHC class I loci, 41 C-type lectin-like loci, and other loci amid hundreds of transposable elements
Source: G3 (Bethesda). 2022 Aug 23;12(11):jkac218. doi: 10.1093/g3journal/jkac218 (PMC9635633; doi:10.1093/g3journal/jkac218)
Supplement: jkac218_Supplementary_Methods [file jkac218_supplementary_methods.pdf]

Supplemental Methods

Preparation of BAC DNA and sequencing

Highly purified BAC DNA was randomly fragmented in g-tubes (Covaris). Following the selection for 20-kb templates in the BluePippin size selection system (Sage Science, Beverly, MA), SMRTbell libraries were constructed according to the PacBio protocol. After construction, libraries were size selected again. Sequencing primers were annealed, and nucleotides were incorporated by polymerase bound on the hairpin loops of the dumbbell-shaped templates. Templates were complexed with the PacBio MagBeads. The template-MagBeads complexes were loaded on the PacBio RS II Sequencer. Primary analyses, including images in real-time, base calling and quality assessment, were processed through the PacBio RS Blade Center using RS Touch and RS Remote.

Sequence assembly for individual BAC clones

For all assemblies, care was taken in making linear representations of the sequences of the circular BAC constructs. This includes identifying and removing the BAC backbone. Briefly, sequences based upon Hierarchical Genome Assembly Process v3 (HGAP3) assemblies were used for 19d16, 58f18, and 34j16. For all assemblies, the RS Blade Center was used to produce raw .bax.h5 files. When HGAP3 was used for the initial assembly sequence, primary SMRT sequencing data were streamed directly into the SMRT Pipe (v1.87.139483) via SMRT Portal (v2.3.0). Canu (Koren et al. 2017) was used for other clones. Canu v1.5 were used for 102b15 and 190m7. Canu v2.1 assemblies were used for 1o23 and 173o1. For Canu assemblies, raw .bax.h5 files were converted to an intermediate bam file using `bax2bam` (https://github.com/PacificBiosciences/pbbioconda) and subread FASTQ files were then created using samtools (v1.9) 'mkfastq' (Li et al. 2009). Subreads greater than 10,000 bp were used for Canu assemblies. Additional details are provided on GitHub: https://github.com/cwarden45/Miller_Red_Jungle_Fowl_MHCY/blob/main/Part1_Assembly/README.md.

Assemblies were selected that had relatively uniform subread coverage. Additional small-scale polishing was performed to improve the sequence quality. HGAP3 assemblies were polished first with Quiver. For all assemblies, subread alignments were performed with BLASR (Basic Local Alignment with Successive Refinement (Chaisson and Tesler 2012) and polishing was then performed with Arrow. The PacBio implementation for Arrow and BLASR in pbbioconda (v2.3.3 and v5.3.3 respectively) was used for these steps.

For some assemblies, alignments were further evaluated with PacBio circular consensus sequencing (CCS) HiFi reads and with Illumina sequence data. For 190m7 and 58f18, 10X CCS reads were generated using the `ccs` function in the unanimity package (https://hub.docker.com/r/cwarden45/general-pacbio/). The samtools (v1.9) 'bam2fq' function was used to create CCS FASTQ files. Illumina sequence data was available for 190m7, 173o1, and 34j16. BWA-MEM (v0.7.15-r1140) (Li 2013) was used to align PacBio CCS and Illumina reads to each other and to the assembled BAC sequences. Reads were sorted and read groups were defined with Picard AddOrReplaceReadGroups (https://broadinstitute.github.io/picard/, v2.21.1). Duplicate reads were removed using Picard MarkDuplicates (https://broadinstitute.github.io/picard/, v2.21.1). A .pileup file with the "-C50" parameter was created using samtools mpileup v1.4(Li et al. 2009). VarScan v2.3.9 (Koboldt et al. 2012) was used to call variants using a conservative set of parameters (Warden et al. 2014). When available, we compared data from both Illumina and PacBio CCS libraries; we found no

common homozygous variants and made no additional changes in the MHCY region. The polished 34j16 sequence was identical to the previous Sanger assembly. No changes were made to 34j16. The 173o1 sequence was compared to J_AA173O01 in AC275299.1 and to an earlier Sanger sequence assembly. Three changes were made to 173o1: T→TC at 31505, C→CG at 45669, C→CG at 124293 (the numbers indicate the positions prior to correction). Overall, there was little evidence for changes within the MHCY region of the assembly based on variant calls. More details can be found at: https://github.com/cwarden45/Miller_Red_Jungle_Fowl_MHCY/tree/main/Part1_Assembly.

Assembly of Contig 1

Following a "working draft" assembly, 190m7 and 19d16 sequence were combined (with the internal, common *Eco*RI site overlapped). The five clones (173o1, 102b15, 1o23, 190m7, and 19d16) were then compared to this initial sequence representing the entire length of Contig 1. BLAST ((Altschul et al. 1990), blastn v2.2.27+, parameters "-perc_identity 95 -evalue 1e-20") was used to confirm the relationships between the contigs and individual clones without identifying sequence to change within Contig 1. In addition, the five separate clones were aligned to the draft Contig 1 sequence using BWA-MEM v 0.7.15-r1140 (Li 2013), with the "-M" parameter. BWA-MEM can split sequences to create supplemental reads. Initial inspection of supplemental BWA-MEM alignments for 1o23 and 102b15 (the two clones other than 190m7 that contained NOR sequence), showed some noticeable divergence suggesting that these two sequences might be incorrectly aligned. Those alignments were manually removed so the resulting BWA-MEM alignment was in relatively good concordance to Contig 1 for each clone. Variations from the draft reference sequence in the BWA-MEM alignment were summarized by creating a .pileup file (using samtools v1.4 (Li et al. 2009) with default parameters).

Seven changes were made to the 190m7 sequence where the consensus of overlapping sequences suggested there were errors: C→ CG at 95439, GC→G at 101375, AC→A at 103583, A → AT at 103685, A → AC at 103997, A → AC at 104654, and T → TC at 104976 (the numbers indicate the positions prior to correction). In the area where only 19d16 and 173o1 overlapped, nucleotide mismatches could not be resolved by majority calls. For mismatches in this region, polished calls were used to determine which call to select. Two additional changes were made to produce the Contig 1 sequence: C → CA and C → CG at previous positions 146229 and 166681, respectively. An additional modification to the Contig 1 consensus sequence was made later by adding a five bp repeat (AAGGG) at position 339,990. The code associated with this assembly is available: https://github.com/cwarden45/Miller_Red_Jungle_Fowl_MHCY/tree/main/Part1_Assembly/Contig1

Definition of Contig 2 and Contig 3

Contig 2 and Contig 3 are the sequences of single clones, 58f18 and 34j16 respectively. While they have sequences to each other and to Contigs 1 and 4, the sequence could not be assembled into a single sequence.

Assembly of Contig 4 from fosmid clones

Also included in this study are GenBank sequences for two fosmid clones: AC270441.1 (clone J_AD-484G5) and AC270418.1 (clone J_AE-174A8) from UCD001 #256 fosmid libraries. These were deposited in GenBank by others (Bellott et al. 2017), but not assigned to a chromosome. They were found in searches of GenBank to have high-quality matches to the MHCY BAC sequences. They form Contig 4 (45,013 bp) in which there is a 27,060 bp 100% identity overlap between the two clones. Only two modifications were made to produce Contig

4. These were the removal of one mismatching nucleotide from the end of AC270418.1 in the region of overlap and the removal of 25 bp of vector sequence at the opposite end (outside the region of overlap).

Annotations of genes and repeats

Early annotations were generated by running GENSCAN (Burge and Karlin 1997) on unmasked contig sequences. Annotation of each predicted locus was critically assessed. This includes visualization of gene expression data and/or additional gene prediction programs (i.e., Exonerate v2.2.0 (Slater and Birney 2005), MAKER v2.31.9 (Holt and Yandell 2011), etc.). For OZFL, CPAT (Wang et al. 2013) was used. Additional information is available at https://github.com/cwarden45/Miller_Red_Jungle_Fowl_MHCY/tree/main/Part2_Annotation/Gene_Annotation_Iterations/Additional_Revision_Details.

Ribosomal rRNA gene clusters and individual genes were annotated with BLAST v2.6.0+ (Altschul et al. 1990) using KT445934 as the query sequence (Dyomin et al. 2016).

Two repeat programs were used to annotate repeats. Transposable elements and other repeats were annotated with RepeatMasker v4.0.6 (http://www.repeatmasker.org) using chicken RepBase annotations (Bao et al. 2015) downloaded 4/6/2017 and rmblast v2.2.27 as the search engine and parameters "*-species chicken -gff*"). Tandem repeats were annotated with Tandem Repeat Finder (TRF) , version 4.09 with parameters "*2 7 7 80 10 50 50 -d -m*") (Benson 1999),. Code related to the repeat annotations is available (https://github.com/cwarden45/Miller_Red_Jungle_Fowl_MHCY/tree/main/Part2_Annotation/Repeat_Annotations).

Alignment of Illumina RNA-Seq data and NCBI EST data

To prepare a sequence set suitable for screening RNA-Seq data for evidence of gene expression and to evaluate intron/exon boundaries, a specialized sequence was prepared starting with the galGal5 reference genome. This galGal5 reference sequence was downloaded from the UCSC Genome Browser (Kent et al. 2002) on 5/12/2016. Sequence for GGA16 was modified to remove sequence containing the MHCY region loci identified in galGal5 (all sequence after 350,000 was removed), and the four contig sequences were added in. The goal was to have a genomic sequence data set against which expression data could be compared so that matches within the four contigs would be distinguished from background matches to other regions of the genome.

Illumina paired-end reads from the Chickspress project ((McCarthy et al. 2019), PRJNA204941) were aligned to this custom chicken reference using STAR v2.5version 2.5, with parameters "--*alignIntronMax 2000 --alignSJoverhangMin 20 --alignSJDBoverhangMin 20 --outFilterMismatchNoverLmax 0.1 --outFilterIntronMotifs RemoveNoncanonical --outSAMstrandField intronMotif*" (Dobin et al. 2013). SAM-to-BAM conversion, co-ordinate sorting, and filtering of reads that were aligned to at least one contig sequence were performed using samtools view and indexed (samtools, version 1.6). Additional details related to alignment and quantification of gene expression data can be found here: https://github.com/cwarden45/Miller_Red_Jungle_Fowl_MHCY/tree/main/Part2_Annotation/Alignment_and_Quantification_for_Expression.

Also included on GitHub are additional measures used for visual inspection, including Integrative Genomics Viewer (IGV) (Robinson et al. 2011) that were not directly used for quantification. A variety of parameters were tested with STAR (https://github.com/cwarden45/Miller_Red_Jungle_Fowl_MHCY/discussions/2). TopHat2v2.1.1 (Kim et al. 2013, v2.1.1, v2.1.1, v2.1.1, v2.1.1, v2.1.1, v2.1.1, v2.1.1, v2.1.1) and

STAR alignments were used for visualization of Illumina RNA-Seq data at various stages of assembly and alignment revisions. GMAP v 2018.05.11(Wu and Watanabe 2005) was used to align longer EST sequences downloaded from NCBI Nucleotide.

Assessment of gene annotations using Illumina RNA-Seq data

We use normalized coverage and absolute coverage for all splice junctions in a gene model to assess the gene candidate annotations. For this, the total unique and multi-mapped reads were parsed from the STAR summary files (with extension *_Log.final.out*). These values were used to calculate Count-Per-Million (CPM) normalized coverage for each junction. The raw unique and multi-mapped junction counts were collected from STAR (from files with extension *_SJ.out.tab*). The multi-mapped coverage was adjusted based upon the total number of known copies present in Contigs 1-4. In Table S2, the separately adjusted total CPM values were summed across copies of 100% identical genes for each unique gene sequence.

If all junctions in the gene had at least one hundred reads in at least ten libraries, the gene was assigned to evidence category "Good". If all junctions in the gene had at least ten reads in at least three libraries, the gene was assigned to evidence category "Intermediate". Genes with at least one read in at least one sample for all junctions were assigned to evidence level "Low". If there was not read evidence for all junctions with the main set of STAR alignment parameters used, then we inspected the alignments and decided if the gene should be kept. Rather than using "pseudogene" to describe sequences with very low (one block) scores, an evidence level of "Poor" was assigned. Intermediate tables and the code to define gene annotation confidence using the STAR splice junctions from Illumina RNA-Seq data can be found here: https://github.com/cwarden45/Miller_Red_Jungle_Fowl_MHCY/tree/main/Part2_Annotation/STAR_Splice_Junction_Evidence. There is also an additional strategy to quantify the evenness of expression that complements the assignments for all "Good" evidence genes as well as a selected number of "Intermediate" evidence genes (https://github.com/cwarden45/Miller_Red_Jungle_Fowl_MHCY/discussions/3).

dN/dS Divergence Methods

All MHCY sequences are provided from this paper, and analysis uses MHCY sequences shown in Figure S3. For the HLA comparison, a list of 12 from HLA-A and 13 from HLA-B alleles was used to find representative sequences in IMGT/HLA (Robinson et al. 2020). A representative sequence from GenBank was selected based upon certain criteria, and it was confirmed that the nucleotide sequence for that specific GenBank entry was 100% match to what is provided by IMGT/HLA (and provided allele information in the GenBank deposit was compatible with IMGT/HLA). If needed, Biopython (Cock et al. 2009)was used to extract coding (CDS) sequences from GenBank deposits. Likewise, as needed, the "Translate tool" from ExPASy was used for manual sequence inspection (Gasteiger et al. 2003).

A neighbor joining tree (Saitou and Nei 1987) of peptide alignments for the α1and α2 domains were created using Clustal Omega (Sievers et al. 2011; Goujon et al. 2010). Phylogenetic trees in text format were exported from Clustal Omega and a scaled tree for amino acid divergence was created using ggtree (Yu et al. 2017).
Divergence values (dN and dS) were calculated using codeml in PAML (v4.8, (Yang 2007)), using the Nei and Gojobori (Nei and Gojobori 1986) estimates. The configuration file was based upon that provided by Bitarello (Bitarello et al. 2016) for HLA sequences. A modified version of the configuration file and all other code necessary to reproduce results are available at https://github.com/cwarden45/Miller_Red_Jungle_Fowl_MHCY/tree/main/Part2_Annotation/dN_dS_Analysis.

The input to PAML was created using PAL2NAL (Suyama et al. 2006), which uses Clustal Omega (Sievers et al. 2011; Goujon et al. 2010) protein alignments with the similarity row removed (along with a FASTA file of paired nucleotide sequences). The option to remove gapped alignment sequences in PAL2NAL was selected.

Literature Cited

Altschul, S.F., W. Gish, W. Miller, E.W. Myers, and D.J. Lipman, 1990 Basic local alignment search tool. *J Mol Biol* 215 (3):403-410.

Bao, W., K.K. Kojima, and O. Kohany, 2015 Repbase Update, a database of repetitive elements in eukaryotic genomes. *Mob DNA* 6:11.

Bellott, D.W., H. Skaletsky, T.J. Cho, L. Brown, D. Locke *et al.*, 2017 Avian W and mammalian Y chromosomes convergently retained dosage-sensitive regulators. *Nat Genet* 49 (3):387-394.

Benson, G., 1999 Tandem repeats finder: a program to analyze DNA sequences. *Nucleic Acids Res* 27 (2):573-580.

Bitarello, B.D., S. Francisco Rdos, and D. Meyer, 2016 Heterogeneity of dN/dS Ratios at the Classical HLA Class I Genes over Divergence Time and Across the Allelic Phylogeny. *J Mol Evol* 82 (1):38-50.

Burge, C., and S. Karlin, 1997 Prediction of complete gene structures in human genomic DNA. *J Mol Biol* 268 (1):78-94.

Chaisson, M.J., and G. Tesler, 2012 Mapping single molecule sequencing reads using basic local alignment with successive refinement (BLASR): application and theory. *BMC Bioinformatics* 13:238.

Cock, P.J., T. Antao, J.T. Chang, B.A. Chapman, C.J. Cox *et al.*, 2009 Biopython: freely available Python tools for computational molecular biology and bioinformatics. *Bioinformatics* 25 (11):1422-1423.

Dobin, A., C.A. Davis, F. Schlesinger, J. Drenkow, C. Zaleski *et al.*, 2013 STAR: ultrafast universal RNA-seq aligner. *Bioinformatics* 29 (1):15-21.

Dyomin, A.G., E.I. Koshel, A.M. Kiselev, A.F. Saifitdinova, S.A. Galkina *et al.*, 2016 Chicken rRNA Gene Cluster Structure. *PLoS One* 11 (6):e0157464.

Gasteiger, E., A. Gattiker, C. Hoogland, I. Ivanyi, R.D. Appel *et al.*, 2003 ExPASy: The proteomics server for in-depth protein knowledge and analysis. *Nucleic Acids Res* 31 (13):3784-3788.

Goujon, M., H. McWilliam, W. Li, F. Valentin, S. Squizzato *et al.*, 2010 A new bioinformatics analysis tools framework at EMBL-EBI. *Nucleic Acids Res* 38 (Web Server issue):W695-699.

Holt, C., and M. Yandell, 2011 MAKER2: an annotation pipeline and genome-database management tool for second-generation genome projects. *BMC Bioinformatics* 12:491.

Kent, W.J., C.W. Sugnet, T.S. Furey, K.M. Roskin, T.H. Pringle *et al.*, 2002 The human genome browser at UCSC. *Genome Res* 12 (6):996-1006.

Kim, D., G. Pertea, C. Trapnell, H. Pimentel, R. Kelley *et al.*, 2013 TopHat2: accurate alignment of transcriptomes in the presence of insertions, deletions and gene fusions. *Genome Biol* 14 (4):R36.

Koboldt, D.C., Q. Zhang, D.E. Larson, D. Shen, M.D. McLellan *et al.*, 2012 VarScan 2: somatic mutation and copy number alteration discovery in cancer by exome sequencing. *Genome Res* 22 (3):568-576.

Koren, S., B.P. Walenz, K. Berlin, J.R. Miller, N.H. Bergman *et al.*, 2017 Canu: scalable and accurate long-read assembly via adaptive k-mer weighting and repeat separation. *Genome Res* 27 (5):722-736.

Li, H., 2013 Aligning sequence reads, clone sequences and assembly contigs with BWA-MEM, pp. p. 1303.1399, arXiv

Li, H., B. Handsaker, A. Wysoker, T. Fennell, J. Ruan *et al.*, 2009 The Sequence Alignment/Map format and SAMtools. *Bioinformatics* 25 (16):2078-2079.

McCarthy, F.M., K. Pendarvis, A.M. Cooksey, C.R. Gresham, M. Bomhoff *et al.*, 2019 Chickspress: a resource for chicken gene expression. *Database (Oxford)* 2019.

Nei, M., and T. Gojobori, 1986 Simple methods for estimating the numbers of synonymous and nonsynonymous nucleotide substitutions. *Mol Biol Evol* 3 (5):418-426.

Robinson, J., D.J. Barker, X. Georgiou, M.A. Cooper, P. Flicek *et al.*, 2020 IPD-IMGT/HLA Database. *Nucleic Acids Res* 48 (D1):D948-d955.

Robinson, J.T., H. Thorvaldsdóttir, W. Winckler, M. Guttman, E.S. Lander *et al.*, 2011 Integrative genomics viewer. *Nat Biotechnol* 29 (1):24-26.

Saitou, N., and M. Nei, 1987 The neighbor-joining method: a new method for reconstructing phylogenetic trees. *Mol Biol Evol* 4 (4):406-425.

Sievers, F., A. Wilm, D. Dineen, T.J. Gibson, K. Karplus *et al.*, 2011 Fast, scalable generation of high-quality protein multiple sequence alignments using Clustal Omega. *Mol Syst Biol* 7:539.

Slater, G.S., and E. Birney, 2005 Automated generation of heuristics for biological sequence comparison. *BMC Bioinformatics* 6:31.

Suyama, M., D. Torrents, and P. Bork, 2006 PAL2NAL: robust conversion of protein sequence alignments into the corresponding codon alignments. *Nucleic Acids Res* 34 (Web Server issue):W609-612.

Wang, L., H.J. Park, S. Dasari, S. Wang, J.P. Kocher *et al.*, 2013 CPAT: Coding-Potential Assessment Tool using an alignment-free logistic regression model. *Nucleic Acids Res* 41 (6):e74.

Warden, C.D., A.W. Adamson, S.L. Neuhausen, and X. Wu, 2014 Detailed comparison of two popular variant calling packages for exome and targeted exon studies. *PeerJ* 2:e600.

Wu, T.D., and C.K. Watanabe, 2005 GMAP: a genomic mapping and alignment program for mRNA and EST sequences. *Bioinformatics* 21 (9):1859-1875.

Yang, Z., 2007 PAML 4: phylogenetic analysis by maximum likelihood. *Mol Biol Evol* 24 (8):1586-1591.

Yu, L., Y. Zhang, I. Gutman, Y. Shi, and M. Dehmer, 2017 Protein Sequence Comparison Based on Physicochemical Properties and the Position-Feature Energy Matrix. *Sci Rep* 7:46237.
