# Supplementary material for: The Gallus gallus RJF reference genome reveals an MHCY haplotype organized in gene blocks that contain 107 loci including 45 specialized, polymorphic MHC class I loci, 41 C-type lectin-like loci, and other loci amid hundreds of transposable elements
Source: G3 (Bethesda). 2022 Aug 23;12(11):jkac218. doi: 10.1093/g3journal/jkac218 (PMC9635633; doi:10.1093/g3journal/jkac218)
Supplement: jkac218_Supplementary_Tables_S1-S3 [file jkac218_supplementary_tables_s1-s3.pdf]

Table S1. List of all rRNA and MHCY genes within the four RJF contigs.

Gene List No.	Contig	Contig Gene No.	Location (bp)	Length (bp)	Strand	Number Exons Predicted	Region	Gene Symbol	Gene Type	Locus Tag
1	1	1	35177-47027	11850	+	na	NOR	rRNA	rRNA	RJF.Contig1.LOC01
2	1	2	60631-72501	11870	+	na	NOR	rRNA	rRNA	RJF.Contig1.LOC02
3	1	3	91569-103436	11867	+	na	NOR	rRNA	rRNA	RJF.Contig1.LOC03
4	1	4	107423-107936	514	-	ps*	MHCY	YLEC1P	YLEC-ps	RJF.Contig1.LOC04.YLEC1P
5	1	5	122604-124022	1419	+	NA	MHCY	OZFL	OZFL	RJF.Contig1.LOC05.OZFL
6	1	6	127020-128486	870	+	6	MHCY	MHCY2B1	MHCY2B-a	RJF.Contig1.LOC06.MHCY2B1
7	1	7	133428-135769	1428	-	4	MHCY	ZNFY1	ZNFY-a	RJF.Contig1.LOC07.ZNFY1
8	1	8	141328-141435	108	+	ps	MHCY	YLEC2P	YLEC-ps	RJF.Contig1.LOC08.YLEC2P
9	1	9	147289-147436	148	-	ps	MHCY	YLEC3P	YLEC-ps	RJF.Contig1.LOC09.YLEC3P
10	1	10	153041-153147	107	-	ps	MHCY	YLEC4P	YLEC-ps	RJF.Contig1.LOC10.YLEC4P
11	1	11	158707-161048	1428	+	4	MHCY	ZNFY2	ZNFY-a	RJF.Contig1.LOC11.ZNFY2
12	1	12	165996-167463	870	-	6	MHCY	MHCY2B2	MHCY2B-b	RJF.Contig1.LOC12.MHCY2B2
13	1	13	174026-176058	1062	-	8	MHCY	MHCY1	MHCY-d	RJF.Contig1.LOC13.MHCY1
14	1	14	186371-188030	999	-	6	MHCY	MHCY2	MHCY-v	RJF.Contig1.LOC14.MHCY2
15	1	15	188744-189609	866	+	ps	MHCY	YLEC5P	YLEC-ps	RJF.Contig1.LOC15.YLEC5P
16	1	16	191549-193595	1062	-	8	MHCY	MHCY3	MHCY-h	RJF.Contig1.LOC16.MHCY3
17	1	17	204195-205854	999	-	6	MHCY	MHCY4	MHCY-v	RJF.Contig1.LOC17.MHCY4
18	1	18	206963-207433	471	+	ps	MHCY	YLEC6P	YLEC-ps	RJF.Contig1.LOC18.YLEC6P
19	1	19	209373-211433	1062	-	8	MHCY	MHCY5	MHCY-f	RJF.Contig1.LOC19.MHCY5
20	1	20	216901-218550	1650	+	ps	MHCY	YLEC7P	YLEC-ps	RJF.Contig1.LOC20.YLEC7P
21	1	21	222883-224477	1047	+	5	MHCY	MHCY6	MHCY-u	RJF.Contig1.LOC21.MHCY6
22	1	22	227492-229463	831	-	7	MHCY	YLEC8	YLEC-a	RJF.Contig1.LOC22.YLEC8
23	1	23	230446-232427	831	-	7	MHCY	YLEC9	YLEC-b	RJF.Contig1.LOC23.YLEC9
24	1	24	233411-235401	831	-	7	MHCY	YLEC10	YLEC-c	RJF.Contig1.LOC24.YLEC10
25	1	25	236748-237872	1125	-	ps	MHCY	YLEC11P	YLEC-ps	RJF.Contig1.LOC25.YLEC11P
26	1	26	239288-241187	1900	-	ps	MHCY	YLEC12P	YLEC-ps	RJF.Contig1.LOC26.YLEC12P
27	1	27	243737-245613	1035	+	7	MHCY	MHCY7	MHCY-l	RJF.Contig1.LOC27.MHCY7
28	1	28	257602-257802	201	+	ps	MHCY	MHCY2B3P	MHCY2B-ps	RJF.Contig1.LOC28.MHCY2B3P
29	1	29	258475-259160	686	+	ps	MHCY	LENG9L1P	LENG9L-ps	RJF.Contig1.LOC29.LENG9L1P
30	1	30	262966-265017	1062	-	8	MHCY	MHCY8	MHCY-a	RJF.Contig1.LOC30.MHCY8
31	1	31	268634-270623	828	+	7	MHCY	YLEC13	YLEC-d	RJF.Contig1.LOC31.YLEC13
32	1	32	273619-275680	1062	-	8	MHCY	MHCY9	MHCY-i	RJF.Contig1.LOC32.MHCY9
33	1	33	285736-287369	981	-	6	MHCY	MHCY10	MHCY-t	RJF.Contig1.LOC33.MHCY10
34	1	34	297834-299776	1125	-	7	MHCY	MHCY11	MHCY-o	RJF.Contig1.LOC34.MHCY11
35	1	35	302271-302635	365	+	ps	MHCY	YLEC14P	YLEC-ps	RJF.Contig1.LOC35.YLEC14P
36	1	36	304109-305593	1485	+	ps	MHCY	YLEC15P	YLEC-ps	RJF.Contig1.LOC36.YLEC15P
37	1	37	311376-313367	1173	+	7	MHCY	MHCY12	MHCY-q	RJF.Contig1.LOC37.MHCY12

38	1	38	322029-322209	181	+	ps	MHCY	LENG9L2P	LENG9L-ps	RJF.Contig1.LOC38.LENG9L2P
39	1	39	326000-328067	1062	-	8	MHCY	MHCY13	MHCY-e	RJF.Contig1.LOC39.MHCY13
40	1	40	332501-332865	365	+	ps	MHCY	YLEC16P	YLEC-ps	RJF.Contig1.LOC40.YLEC16P
41	1	41	333848-335829	825	+	7	MHCY	YLEC17	YLEC-e	RJF.Contig1.LOC41.YLEC17
42	1	42	338191-339826	981	-	6	MHCY	MHCY14	MHCY-s	RJF.Contig1.LOC42.MHCY14
43	1	43	349539-351520	828	+	7	MHCY	YLEC18	YLEC-f	RJF.Contig1.LOC43.YLEC18
44	1	44	353195-355037	1056	+	7	MHCY	MHCY15	MHCY-m	RJF.Contig1.LOC44.MHCY15
45	1	45	356555-356866	312	+	ps	MHCY	MHCY16P	MHCY-ps	RJF.Contig1.LOC45.MHCY16P
46	1	46	367726-367926	201	+	ps	MHCY	MHCY2B4P	MHCY2B-ps	RJF.Contig1.LOC46.MHCY2B4P
47	1	47	368600-369286	687	+	NA	MHCY	LENG9L3	LENG9L-a	RJF.Contig1.LOC47.LENG9L3
48	1	48	373092-375143	1062	-	8	MHCY	MHCY17	MHCY-a	RJF.Contig1.LOC48.MHCY17
49	1	49	378760-380749	828	+	7	MHCY	YLEC19	YLEC-d	RJF.Contig1.LOC49.YLEC19
50	1	50	383745-385806	1062	-	8	MHCY	MHCY18	MHCY-i	RJF.Contig1.LOC50.MHCY18
51	1	51	395862-397495	981	-	6	MHCY	MHCY19	MHCY-t	RJF.Contig1.LOC51.MHCY19
52	1	52	407960-409902	1125	-	7	MHCY	MHCY20	MHCY-o	RJF.Contig1.LOC52.MHCY20
53	2	1	894-1073	180	+	ps	MHCY	LENG9L4P	LENG9L-ps	RJF.Contig2.LOC01.LENG9L4P
54	2	2	4870-6937	1062	-	8	MHCY	MHCY21	MHCY-e	RJF.Contig2.LOC02.MHCY21
55	2	3	11371-11735	365	+	ps	MHCY	YLEC20P	YLEC-ps	RJF.Contig2.LOC03.YLEC20P
56	2	4	12718-14698	825	+	7	MHCY	YLEC21	YLEC-e	RJF.Contig2.LOC04.YLEC21
57	2	5	17060-18695	981	-	6	MHCY	MHCY22	MHCY-s	RJF.Contig2.LOC05.MHCY22
58	2	6	28416-30397	828	+	7	MHCY	YLEC22	YLEC-f	RJF.Contig2.LOC06.YLEC22
59	2	7	32072-33914	1056	+	7	MHCY	MHCY23	MHCY-m	RJF.Contig2.LOC07.MHCY23
60	2	8	35432-35743	312	-	ps	MHCY	MHCY24P	MHCY-ps	RJF.Contig2.LOC08.MHCY24P
61	2	9	46603-46803	201	+	ps	MHCY	MHCY2B5P	MHCY2B-ps	RJF.Contig2.LOC09.MHCY2B5P
62	2	10	47477-48163	687	+	NA	MHCY	LENG9L5	LENG9L-a	RJF.Contig2.LOC10.LENG9L5
63	2	11	51969-54020	1062	-	8	MHCY	MHCY25	MHCY-a	RJF.Contig2.LOC11.MHCY25
64	2	12	57637-59626	828	+	7	MHCY	YLEC23	YLEC-g	RJF.Contig2.LOC12.YLEC23
65	2	13	62622-64683	1062	-	8	MHCY	MHCY26	MHCY-i	RJF.Contig2.LOC13.MHCY26
66	2	14	74739-76372	981	-	6	MHCY	MHCY27	MHCY-t	RJF.Contig2.LOC14.MHCY27
67	2	15	86832-88774	1125	-	7	MHCY	MHCY28	MHCY-o	RJF.Contig2.LOC15.MHCY28
68	2	16	99257-100916	999	-	6	MHCY	MHCY29	MHCY-v	RJF.Contig2.LOC16.MHCY29
69	2	17	101759-102495	737	+	ps	MHCY	YLEC24P	YLEC-ps	RJF.Contig2.LOC17.YLEC24P
70	2	18	104435-106481	1062	-	8	MHCY	MHCY30	MHCY-h	RJF.Contig2.LOC18.MHCY30
71	2	19	117051-118710	999	-	6	MHCY	MHCY31	MHCY-v	RJF.Contig2.LOC19.MHCY31
72	2	20	119553-120289	737	+	ps	MHCY	YLEC25P	YLEC-ps	RJF.Contig2.LOC20.YLEC25P
73	2	21	122229-124289	1062	-	8	MHCY	MHCY32	MHCY-g	RJF.Contig2.LOC21.MHCY32
74	2	22	129929-131422	1494	-	ps	MHCY	YLEC26P	YLEC-ps	RJF.Contig2.LOC22.YLEC26P
75	2	23	135755-137349	1047	+	5	MHCY	MHCY33	MHCY-u	RJF.Contig2.LOC23.MHCY33
76	2	24	140364-142335	831	-	7	MHCY	YLEC27	YLEC-a	RJF.Contig2.LOC24.YLEC27
77	2	25	143318-145299	831	-	7	MHCY	YLEC28	YLEC-b	RJF.Contig2.LOC25.YLEC28
78	2	26	146283-148273	831	-	7	MHCY	YLEC29	YLEC-c	RJF.Contig2.LOC26.YLEC29

79	3	1	109-580	249	+	2	MHCY	YLEC30partial	na	RJF.Contig3.LOC01.YLEC30partial
80	3	2	5855-7916	1062	+	8	MHCY	MHCY34	MHCY-b	RJF.Contig3.LOC02.MHCY34
81	3	3	14880-15943	1064	+	ps	MHCY	MHCY2B6P	MHCY2B-ps	RJF.Contig3.LOC03.MHCY2B6P
82	3	4	20987-23328	1428	-	4	MHCY	ZNFY3	ZNFY-b	RJF.Contig3.LOC04.ZNFY3
83	3	5	27909-28480	572	+	ps	MHCY	YLEC31P	YLEC-ps	RJF.Contig3.LOC05.YLEC31P
84	3	6	34057-34204	148	-	ps	MHCY	YLEC32P	YLEC-ps	RJF.Contig3.LOC06.YLEC32P
85	3	7	39735-39944	210	-	ps	MHCY	YLEC33P	YLEC-ps	RJF.Contig3.LOC07.YLEC33P
86	3	8	45450-47791	1428	+	4	MHCY	ZNFY4	ZNFY-a	RJF.Contig3.LOC08.ZNFY4
87	3	9	52718-54183	870	-	6	MHCY	MHCY2B7	MHCY2B-c	RJF.Contig3.LOC09.MHCY2B7
88	3	10	61594-63481	1035	-	7	MHCY	MHCY35	MHCY-k	RJF.Contig3.LOC10.MHCY35
89	3	11	65551-67532	831	+	7	MHCY	YLEC34	YLEC-h	RJF.Contig3.LOC11.YLEC34
90	3	12	69008-70498	1491	+	ps	MHCY	YLEC35P	YLEC-ps	RJF.Contig3.LOC12.YLEC35P
91	3	13	75831-77974	1326	+	7	MHCY	MHCY36	MHCY-w	RJF.Contig3.LOC13.MHCY36
92	3	14	87969-90027	1062	+	8	MHCY	MHCY37	MHCY-c	RJF.Contig3.LOC14.MHCY37
93	3	15	93114-93295	182	+	ps	MHCY	LENG9L6P	LENG9L-ps	RJF.Contig3.LOC15.LENG9L6P
94	3	16	101615-103648	1062	-	8	MHCY	MHCY38	MHCY-j	RJF.Contig3.LOC16.MHCY38
95	3	17	109510-109981	472	-	ps	MHCY	YLEC36P	YLEC-ps	RJF.Contig3.LOC17.YLEC36P
96	3	18	111079-111894	444	+	3	MHCY	MHCY39	MHCY-r	RJF.Contig3.LOC18.MHCY39
97	3	19	116973-117659	687	-	NA	MHCY	LENG9L7	LENG9L-b	RJF.Contig3.LOC19.LENG9L7
98	3	20	118345-118545	201	+	ps	MHCY	MHCY2B8P	MHCY2B-ps	RJF.Contig3.LOC20.MHCY2B8P
99	3	21	130265-132141	1035	-	7	MHCY	MHCY40	MHCY-l	RJF.Contig3.LOC21.MHCY40
100	3	22	134691-136590	1900	+	ps	MHCY	YLEC37P	YLEC-ps	RJF.Contig3.LOC22.YLEC37P
101	3	23	138006-138761	756	+	ps	MHCY	YLEC38P	YLEC-ps	RJF.Contig3.LOC23.YLEC38P
102	4	1	6453-6633	181	+	ps	MHCY	LENG9L8P	LENG9L-ps	RJF.Contig4.LOC01.LENG9L8P
103	4	2	10424-12491	1062	-	8	MHCY	MHCY41	MHCY-e	RJF.Contig4.LOC02.MHCY41
104	4	3	16925-17284	360	+	ps	MHCY	YLEC39P	YLEC-ps	RJF.Contig4.LOC03.YLEC39P
105	4	4	18272-20253	825	+	7	MHCY	YLEC40	YLEC-e	RJF.Contig4.LOC04.YLEC40
106	4	5	22615-24250	981	-	6	MHCY	MHCY42	MHCY-s	RJF.Contig4.LOC05.MHCY42
107	4	6	33793-35774	828	+	7	MHCY	YLEC41	YLEC-f	RJF.Contig4.LOC06.YLEC41
108	4	7	37447-37758	312	+	ps	MHCY	MHCY43P	MHCY-ps	RJF.Contig4.LOC07.MHCY43P
109	4	8	38190-40032	1056	+	7	MHCY	MHCY44	MHCY-n	RJF.Contig4.LOC08.MHCY44
110	4	9	41556-41859	304	-	ps	MHCY	MHCY45P	MHCY-ps	RJF.Contig4.LOC09.MHCY45P

*ps=pseudogene

Table S2. Summary of splice junction analysis using RNA-Seq data.
RJF RNA-Seq data for individual members in the MHCY, YLEC, ZNFY, and MHC2B gene families were analyzed to define expressed genes by evaluating evidence of the presence of full sets of splice junctions. Genes with identical predicted amino acid sequence are grouped into types and their splice junction and expression data are considered together. Findings of full junction coverage (number of junctions covered out of number of junctions predicted for gene) are highlighted in green. The Adjusted Mean for splice junction coverage is also taken into consideration when defining the Evdience Grade. When cDNA clones have been identified, these are noted with the Evidence Grade.

Gene	Sequence Type	Locus/Loci	Count	STAR RJF RNA-Seq Splice Junctions				Evidence Grade**
				Minimum 1 Read, Minimum 1 Sample	Minimum 10 Reads, Minimum 3 Samples	Minimum 100 Reads, Minimum 10 Samples	Adjusted Mean*	
MHCY	MHCY-c	MHCY37	1	7 / 7	7 / 7	7 / 7	15.86	Good (+1 cDNA)
MHCY	MHCY-a	MHCY8, MHCY17, MHCY25	3	7 / 7	7 / 7	7 / 7	14.75	Good (+4 cDNA)
MHCY	MHCY-f	MHCY5	1	7 / 7	7 / 7	5 / 7	14.04	Intermediate
MHCY	MHCY-g	MHCY32	1	7 / 7	7 / 7	5 / 7	14.02	Intermediate
MHCY	MHCY-b	MHCY34	1	7 / 7	7 / 7	5 / 7	4.52	Intermediate (+1 cDNA)
MHCY	MHCY-h	MHCY3, MHCY30	2	7 / 7	7 / 7	3 / 7	7.08	Intermediate
MHCY	MHCY-u	MHCY6, MHCY33	2	4 / 4	4 / 4	2 / 4	5.46	Intermediate
MHCY	MHCY-r	MHCY39	1	2 / 2	2 / 2	1 / 2	5.25	Intermediate
MHCY	MHCY-v	HCY2, MHCY4, MHCY29, MHCY3	4	5 / 5	5 / 5	2 / 5	4.36	Intermediate
MHCY	MHCY-o	MHCY11, MHCY20, MHCY28	3	6 / 6	6 / 6	2 / 6	4.35	Intermediate
MHCY	MHCY-i	MHCY9, MHCY18, MHCY26	3	7 / 7	7 / 7	1 / 7 or 2 / 7	3.11	Intermediate
MHCY	MHCY-j	MHCY38	1	7 / 7	7 / 7	2 / 7	1.65	Intermediate
MHCY	MHCY-d	MHCY1	1	7 / 7	7 / 7	0 / 7	1.04	Intermediate
MHCY	MHCY-l	MHCY7, MHCY40	2	6 / 6	6 / 6	1 / 6	1.89	Intermediate
MHCY	MHCY-k	MHCY35	1	6 / 6	6 / 6	0 / 6	0.78	Intermediate
MHCY	MHCY-e	MHCY13, MHCY21, MHCY41	3	7 / 7	7 / 7	0 / 7	1.38	Low
MHCY	MHCY-t	MHCY10, MHCY19, MHCY27	3	5 / 5	5 / 5	0 / 5	0.15	Low
MHCY	MHCY-s	MHCY14, MHCY22, MHCY42	3	5 / 5	4 / 5 or 3 / 5	2 / 5	6	Low
MHCY	MHCY-q	MHCY12	1	6 / 6	5 / 6	2 / 6	1.42	Low
MHCY	MHCY-m	MHCY15, MHCY23	2	6 / 6	5 / 6	1 / 6	1.01	Low
MHCY	MHCY-n	MHCY44	1	6 / 6	4 / 6	1 / 6	0.99	Low
MHCY	MHCY-w	MHCY36	1	6 / 6	5 / 6	0 / 6	0.75	Low
YLEC	YLEC-a	YLEC8, YLEC27	2	6 / 6	6 / 6	5 / 6	12.56	Intermediate
YLEC	YLEC-b	YLEC9, YLEC28	2	6 / 6	6 / 6	4 / 6	12.08	Intermediate
YLEC	YLEC-c	YLEC10, YLEC29	2	6 / 6	6 / 6	1 / 6	1.48	Intermediate
YLEC	YLEC-h	YLEC34	1	6 / 6	5 / 6	0 / 6	0.43	Low
YLEC	YLEC-d	YLEC13, YLEC19	2	6 / 6	4 / 6	0 / 6	0.2	Low
YLEC	YLEC-g	YLEC23	1	6 / 6	4 / 6	0 / 6	0.19	Low
YLEC	YLEC-f	YLEC18, YLEC22, YLEC41	3	6 / 6	1 / 6	0 / 6	0.08	Low
YLEC	YLEC-e	YLEC17, YLEC21, YLEC40	3	6 / 6	2 / 6	0 / 6	0.06	Low
YLEC	YLEC30partial	YLEC30partial	1	1 / 1	0 / 1	0 / 1	0.03	Low
ZNFY	ZNFY-b	ZNFY3	1	3 / 3	3 / 3	0 / 3	0.58	Low
ZNFY	ZNFY-a	ZNFY1, ZNFY2, ZNFY4	3	3 / 3	3 / 3	0 / 3	0.58	Low
MHCY2B	MHCY2B-b	MHCY2B2	1	5 / 5	0 / 5	0 / 5	0.03	Low
MHCY2B	MHCY2B-c	MHCY2B7	1	5 / 5	0 / 5	0 / 5	0.03	Low
MHCY2B	MHCY2B-a	MHCY2B1	1	4 / 5	0 / 5	0 / 5	0.02	Poor

*Count-Per-Million (CPM) values were calculated for splice junctions based upon unique and multi-mapped reads from STAR. STAR multi-mapped reads were then adjusted for the known number of copies. These were usually similar but not identical for all loci. Total CPM values were calculated by summing the unique reads and the multi-mapped reads (following copy number adjustment). To define one representative number for all 100% identical gene copies, adjusted total CPM values were summed across gene copies.

Evidence Grades are: **Good. All junctions covered with at least one hundred reads in at least ten samples and Mean Adjusted Total Junction CPM is greater than 0.6 (per locus). **Intermediate**. All junctions covered with at least ten reads in at least three samples and Mean Adjusted Total Junction CPM is greater than 0.6 (per locus). **Low**. At least one read in at least one sample for all junctions. **Poor**. At least one criteria for "*Low*" Evidence is not meet. However, upon visual inspection, it was determined to leave the annotation as a gene candidate instead converting to a pseudogene.

Table S3. Calculation of Wu-Kabat variability indices (VI) for residues predicted within the ligand binding groove for 16 MHCY class I loci and 25 HLA loci representing peptide binding groove in classical MHC class I molecules. Positions with variability index values (number of amino acids/frequency) of six or more are considered polymorphic and are highlighted in yellow. Frequency is the number of the most common amino acid/total number amino acids. Positions lacking polymorphism are highlighted in grey. MHCY and HLA sequences are aligned across the table to reflect structurally equivalent positions with blank rows added as needed.

MHCY							HLA						
Residue Number	No. of AA types	AA Types	No. most common AA	Total No.	Frequency	Variability Index	Residue Number	No. of AA types	AA Types	No. most common AA	Total No.	Frequency	Variability Index
1	3	G VW	11	15	0.73	4	1	1	G				
2	3	S LP	10	16	0.63	5	2	1	S				
3	1	H					3	1	H				
4	1	S					4	1	S				
5	1	L					5	1	M				
6	2	R H	13	15	0.87	2	6	1	R				
7	1	Y					7	1	Y				
8	1	F					8	1	F				
9	**4**	**L KMV**	**9**	**15**	**0.60**	**7**	9	**6**	**Y FSTDH**	**10**	**25**	**0.40**	**15**
10	1	T					10	1	T				
11	2	G R	12	15	0.80	3	11	2	S A	18	25	0.72	3
12	1	M					12	2	V M	18	25	0.72	3
13	1	T					13	1	S				
14	1	D					14	1	R				
15	1	P					15	1	P				
16	2	G V	14	15	0.93	2	16	1	G				
17	1	P					17	1	R S	24	25	0.96	1
18	1	G					18	1	G				
19	1	M					19	1	E				
20	1	P					20	1	P				
21	2	R Q	9	15	0.60	3	21	1	R				
22	1	F					22	1	F				
23	1	V					23	1	I				
24	2	I A	14	15	0.93	2	24	3	A ST	17	25	0.68	4
25	1	V					25	1	V				
26	1	G					26	1	G				
27	2	Y C	13	15	0.87	2	27	1	Y				
28	1	V					28	1	V				
29	1	D					29	1	D				
30	2	G D	10	15	0.67	3	30	2	D G	24	25	0.96	2
31	**3**	**K ED**	**6**	**15**	**0.40**	**8**	31	1	T				
32	3	L IN	9	15	0.60	5	32	2	Q L	24	25	0.96	2
33	2	F L	13	15	0.87	2	33	1	F				
34	2	G W	13	15	0.87	2	34	1	V				
35	**4**	**K TNI**	**8**	**15**	**0.53**	**8**	35	1	R				
36	1	Y					36	1	F				
37	3	D NS	11	15	0.73	4	37	1	D				
38	1	S					38	1	S				
39	2	K L	13	15	0.87	2	39	1	D				
40	3	S IG	11	15	0.73	4	40	1	A				
for alignment							41	1	A T	24	25	0.96	2
for alignment							42	1	S				
41	2	R K	13	15	0.87	2	43	2	P Q	13	25	0.52	4
42	**3**	**T WS**	**8**	**15**	**0.53**	**6**	44	2	R K	24	25	0.96	2
43	2	A V	9	15	0.60	3	45	3	M TE	14	25	0.56	5
44	3	Q HR	9	15	0.60	5	46	2	E A	23	25	0.92	2
45	1	P					47	1	P				
46	1	I					48	1	R				
47	3	V AM	11	15	0.73	4	49	1	A				
48	2	E G	13	15	0.87	2	50	1	P				
49	3	M KW	9	15	0.60	5	51	1	W				
50	1	L					52	1	I				
51	1	P					53	1	E				
52	1	Q					54	1	Q				
53	1	E					55	1	E				
54	1	D					56	2	G R	23	25	0.92	2
55	2	Q R	10	15	0.67	3	57	1	P				
56	1	E					58	1	E				
57	1	H					59	1	Y				

58	1	W				
59	3	D AV	8	15	0.53	6
60	4	T VAM	7	15	0.47	9
61	2	Q E	12	15	0.80	3
62	1	T				
63	2	Q K	13	15	0.87	2
64	2	K M	14	15	0.93	2
65	1	A				
66	2	R Q	12	15	0.80	3
67	3	D GE	7	15	0.47	6
68	2	G V	12	15	0.80	3
69	1	E				
70	2	L R	9	15	0.60	3
71	3	D EV	10	15	0.67	5
72	1	F				
73	4	D YCS	6	15	0.40	10
74	4	E WGR	5	15	0.33	12
75	4	F GLN	6	15	0.40	10
76	1	L				
77	5	G SHRN	8	15	0.53	9
78	2	R S	14	15	0.93	2
79	1	L				
80	2	P Q	9	15	0.60	3
81	2	E V	9	15	0.60	3
82	4	R HQC	8	15	0.53	8
83	1	Y				
84	1	N				
85	3	K R I	10	15	0.67	5
86	1	S				
87	2	G K	12	15	0.80	3
for alignment						
88	1	G				
89	1	S				
90	1	H				
91	2	T M	14	15	0.93	2
92	2	L M	12	15	0.80	3
93	1	Q				
94	3	K MR	7	15	0.47	6
95	2	M I	14	15	0.93	2
96	2	F I	12	15	0.80	3
97	1	G				
98	1	C				
99	1	D				
100	1	I				
101	1	L				
102	2	E A	14	15	0.93	2
103	1	D				
104	1	G				
105	1	S				
106	1	I				
for alignment						
107	1	R				
108	1	G				
109	2	Y H	14	15	0.93	2
110	1	D				
111	2	Q K	14	15	0.93	2
112	2	Y D	13	15	0.87	2
113		A				
114		F				
115		D				
116		G				
117	2	R K	14	15	0.93	2
118		D				
119	3	F YH	8	15	0.53	6
120	2	L I	8	15	0.53	4
121	1	A				
122	1	F				
123	1	D				
124	1	M				
125	2	D Y	14	15	0.93	2
126	1	T				
127	1	M				
128	2	T M	14	15	0.93	2
129	1	F				

60	1	W				
61	1	D				
62	5	R QGEL	13	25	0.52	10
63	3	E NQ	15	25	0.60	5
64	1	T				
65	3	Q R G	12	25	0.48	6
66	3	I NK	12	25	0.48	6
67	6	V CFSMY	11	25	0.44	14
68	1	K				
69	2	A T	15	25	0.60	3
70	5	N HQSK	10	25	0.40	13
71	3	S TA	12	25	0.48	6
72	1	Q				
73	2	T I	24	25	0.96	2
74	3	D YH	15	25	0.60	5
75	1	R				
76	3	E VA	16	25	0.64	5
77	3	N DS	10	25	0.40	8
78	1	L				
79	2	R G	16	25	0.64	3
80	3	T IN	11	25	0.44	7
81	2	L A	17	25	0.68	3
82	2	R L	16	25	0.64	3
83	2	G R	16	25	0.64	3
84	1	Y				
85	1	Y				
86	1	N				
87	1	Q				
88	1	S				
89	1	E				
90	2	A D	22	25	0.88	2
91	1	G				
92	1	S				
93	1	H				
94	2	T I	22	25	0.88	2
95	4	I VLW	13	25	0.52	8
96	1	Q				
97	7	R MITSNW	10	25	0.40	18
98	1	M				
99	3	Y FS	22	25	0.88	3
100	1	G				
101	1	C				
102	2	D H	24	25	0.96	2
103	2	V L	22	25	0.88	2
104	1	G				
105	2	P S	18	25	0.72	3
106	1	D				
107	2	G W	24	25	0.96	2
108	1	R				
109	2	L F	15	25	0.60	3
110	1	L				
111	1	R				
112	1	G				
113	2	Y H	15	25	0.64	3
114	6	N HRDQE	7	25	0.28	21
115	1	Q				
116	6	D YSFHL	9	25	0.36	17
117	1	A				
118	1	Y				
119	1	D				
120	1	G				
121	1	K				
122	1	D				
123	1	Y				
124	1	I				
125	1	A				
126	1	L				
127	2	N K	22	25	0.88	2
128	1	E				
129	1	D				
130	1	L				
131	2	R S	13	25	0.52	4
132	1	S				
133	1	W				

130	1	T				
131	2	A V	14	15	0.93	2
132	1	A				
133	1	D				
134	1	P				
135	1	V				
136	1	A				
137	1	E				
138	1	I				
139	2	T S	13	15	0.87	2
140	1	K				
141	1	R				
142	1	R				
143	2	W R	13	15	0.87	2
144	1	E				
145	2	T E	8	15	0.53	4
146	2	E G	14	15	0.93	2
147	1	G				
148	2	T E	14	15	0.93	2
149	1	Y				
150	1	A				
151	1	E				
152	1	R				
153	1	W				
154	1	K				
155	1	H				
156	1	E				
157	1	L				
158	1	G				
159	2	T N	14	15	0.93	2
160	1	V				
161	1	C				
162	1	V				
163	1	Q				
164	1	N				
165	1	L				
166	1	R				
167	1	R				
168	1	Y				
169	1	L				
170	1	E				
171	1	H				
172	1	G				
173	1	K				
174	1	A				
175	1	A				
176	2	L V	14	15	0.93	2
177	1	K				
178	1	R				
179	2	R T	14	15	0.93	2

134	1	T				
135	1	A				
136	1	A				
137	1	D				
138	2	T M	13	25	0.52	4
139	1	A				
140	1	A				
141	1	Q				
142	2	I T	24	25	0.96	2
143	1	T				
144	2	Q K	20	25	0.80	3
145	3	R H L	23	25	0.92	3
146	1	K				
147	1	W				
148	1	E				
149	2	A T	24	25	0.96	2
150	2	A V	24	25	0.96	2
for alignment						
151	2	R H	19	25	0.76	3
152	3	V AE	16	25	0.64	5
153	1	A				
154	1	E				
155	1	Q				
156	**5**	**L RQWD**	**17**	**25**	**0.68**	**7**
157	1	R				
158	3	A TV	22	25	0.88	3
159	1	Y				
160	1	L				
161	2	E D	24	25	0.96	2
162	1	G				
163	**4**	**T RLE**	**15**	**25**	**0.60**	**7**
164	1	C				
165	1	V				
166	2	E D	22	25	0.88	2
167	2	W G	22	25	0.88	2
168	1	L				
169	1	R				
170	1	R				
171	2	Y H	23	25	0.92	2
172	1	L				
173	1	E				
174	1	N				
175	1	G				
176	1	K				
177	2	E D	24	25	0.96	2
178	1	T				
179	1	L				
180	2	Q E	24	25	0.96	2
181	1	R				
182	2	A T	13	25	0.52	4
